# Pd loaded amphiphilic COF as catalyst for multi-fold Heck reactions, C-C couplings and CO oxidation

**DOI:** 10.1038/srep10876

**Published:** 2015-06-09

**Authors:** Dinesh Mullangi, Shyamapada Nandi, Sorout Shalini, Sheshadri Sreedhala, Chathakudath P. Vinod, Ramanathan Vaidhyanathan

**Affiliations:** 1 Department of Chemistry, Indian Institute of Science Education and Research, Pune, India; 2 CSIR-NCL Catalysis and Inorganic Chemistry Division, Pune, India

## Abstract

COFs represent a class of polymers with designable crystalline structures capable of interacting with active metal nanoparticles to form excellent heterogeneous catalysts. Many valuable ligands/monomers employed in making coordination/organic polymers are prepared via Heck and C-C couplings. Here, we report an amphiphilic triazine COF and the facile single-step loading of Pd^0^ nanoparticles into it. An 18–20% nano-Pd loading gives highly active composite working in open air at low concentrations (Conc. Pd(0) <0.05 mol%, average TON 1500) catalyzing simultaneous multiple site Heck couplings and C-C couplings using ‘non-boronic acid’ substrates, and exhibits good recyclability with no sign of catalyst leaching. As an oxidation catalyst, it shows 100% conversion of CO to CO_2_ at 150 °C with no loss of activity with time and between cycles. Both vapor sorptions and contact angle measurements confirm the amphiphilic character of the COF. DFT-TB studies showed the presence of Pd-triazine and Pd-Schiff bond interactions as being favorable.

Covalent organic frameworks (COFs) are being researched widely as a candidate for applications requiring high surface areas and porosities^1^,^2^,^3^,^4^,^5^,^6^. Condensation reactions between a variety of functional groups have resulted in a number of COFs^7^,^8^,^9^,^10^,^11^,^12^,^13^,^14^,^15^,^16^,^17^,^18^,^19^,^20^,^21^. Some of the earliest reported COFs are the Schiff base ones^22^,^23^,^24^,^25^, formed by reacting amines with aldehyde. They form ideal targets for ready reactions under relatively mild conditions and tend to form well-defined hexagonal honeycomb layers and also high symmetry three dimensional structures depending on the geometry of the building units. Presence of organic backbones in the COFs favor the introduction of specific functionalities by design. Moreover, it has been well demonstrated in coordination chemistry that the imine type (Schiff base) ligands are versatile in incorporating a variety of metal ions^26^,^27^. However, the Schiff bonds need to be proximal to each other for efficient interaction with metals^25^. This requirement poses a constraint in using longer or bulkier molecules in forming the COF, as they would naturally space out the Schiff bonds. There are two solutions to overcome this, one comes from structural part: the bulkier and longer units in the COF could find efficient packing when coupled with flexible functional groups (e.g ether, ester, amide), thus favoring Schiff bonds from adjacent layers to orient proximally. Another is from introducing any N-rich monomers such as triazine derivatives, which could serve as strong interactions sites for the metal nanoparticles. Amorphous polymers built from Triazine cores are known to interact well with catalytically active noble metals, thus preventing catalyst leaching^28^,^29^,^30^,^31^,^32^.

The possibility of forming a variety of products based on a general synthetic methodology has made the metal-catalyzed Heck couplings and boronic acid based C-C bond formations (Suzuki coupling) two of the most important organic reactions^33^,^34^. Polymeric solids based on ion-exchange resins^35^, polystyrene^36^, polymethylacrylate derivatives^37^, covalent organic frameworks (COFs)^38^,^39^,^40^, and hybrid metal–organic frameworks^41^,^42^ have recently been explored as supports for catalytic metal nanoparticles. Yet, the commercial productions still predominantly utilize homogeneous molecular Pd^2+^ based catalysts, owing to their ability to provide non-polar solvent environment required for obtaining products in high yields. In fact, many of the above referred polymers are rich in sp^2^ carbon capable of providing an apolar environment and stabilizing geometries of the catalyst and transition states^7^,^8^,^9^,^10^,^11^,^12^,^13^,^14^,^15^,^16^,^17^,^18^,^19^,^20^,^21^,^35^,^36^,^37^. An inherent compromise that is being made during the use of C-rich supports is the creation of weaker interactions with the catalyst nano-particles, particularly in the case of Pd^0^^43^. This could have immediate implications on the catalyst leaching from the support^44^,^45^. Thus, there is a need for developing heterogeneous catalyst that can provide sp^2^ center rich environments and have strong interactions with both Pd^2+^ and Pd^0^ and at the same time be optimally polar. Stronger Pd^0^-Triazine interactions could be crucial to preventing the Ostwald ripening of the nanoparticles. In fact, triazine based polymer has been shown to interact well with Pd^4^,^29^.

Here, we have formed a Nitrogen rich, triazine based covalent organic framework (trzn-COF) pre-disposed for ‘single-step’ loading of Pd^0^ nanoparticles. Triazine mimics basic pyridyl groups capable of assisting Pd^2+^ to Pd^0^ reduction in an alcoholic medium, and also creates strong interactions with Pd nanoparticles. Additionally, the ether bonds along with the sp^2^ rich non-polar phenyl framework and Schiff linkages serve as an apolar matrix mimicking the environment present typically in homogeneous molecular catalysts^46^. Pd-trzn-COF exhibits good hydrolytic stability, facile handling and recovery and excellent recyclability for a vast library of Heck type reactions and C-C bond formation reactions. Of particular mention is the extremely low catalyst consumption (low amounts of catalyst results in Pd(0) concentrations <0.05 mol% being used in some of the Heck and Suzuki coupling reactions).

Catalytic activity of the trzn-COF has been demonstrated using Heck and C-C coupling reactions^44^. We have chosen the catalysis with a theme of addressing challenges in the areas of Heck couplings and C-C coupling rather than just demonstrating prototype reactions. Specifically, we have demonstrated the simultaneous or multi-fold Heck couplings on a substrate (up to six-fold substitution) which is generally quite difficult to achieve^47^. For the C-C bond formation reactions, homo coupling reactions have been chosen as they represent important non-boronic acid based method to form value-added chemicals. We have used the Pd-trzn-COF as catalyst in the formation of certain organic products which are being sold commercially as excellent ligands or monomers for use in development of advanced materials such as MOFs, coordination complexes and highly functional organic polymers.

Recently COF supported noble metals have been used to carry out industrially important conversions such as methane to methanol^48^, glycerol oxidation^49^, and also as electrocatalysts^4^. To assess the ability of Pd-trzn-COF to mimic a truly industrial catalyst, it was employed as catalyst in the widely studied CO oxidation reaction^50^. This CO to CO_2_ oxidative conversion finds application in air cleaning, automobile catalytic converters, and in cleaning of fuel cell feeds^51^,^52^,^53^. Considering the mere cost of noble metal catalysts still there is a need to bring down the catalyst concentration in reactions and also increase its durability and recyclability. Also, CO needs to interact selectively over O_2_ and H_2_.

## Results

### Preparation and characterization

trzn-COF was synthesized via a solvothermal reaction between 4,4’,4”-(1,3,5-triazine-2,4,6-triyl)tris(oxy))tribenzaldehyde and benzene-1,4-diamine in 1,4 dioxane, mesitylene and aqueous acetic acid by heating at 120 °C for 72 hrs (supporting information). Alternatively, it can be prepared by carrying out the same reaction at room temperature for 12 hrs. The product was isolated as a brown powder by vacuum filtration and washed with different solvents including DMF, THF, DMSO, methanol, water and acetone. Sample from the room temperature synthesis showed better crystallinity, while the high temperature one showed slightly reduced intensities under PXRD, however has much better porosity (~35% increased porosity compared to RT phase). This could point towards the structure obtained under high temperature being favored to crystallize with more ordered pores.

The Pd^0^ was loaded by stirring 100 mg of trzn-COF in an ethanolic solution containing 0.15mmol of Pd(OAc)_2_ at room temperature for 24 hrs.

### Structural solution and description

**Figure 1:**
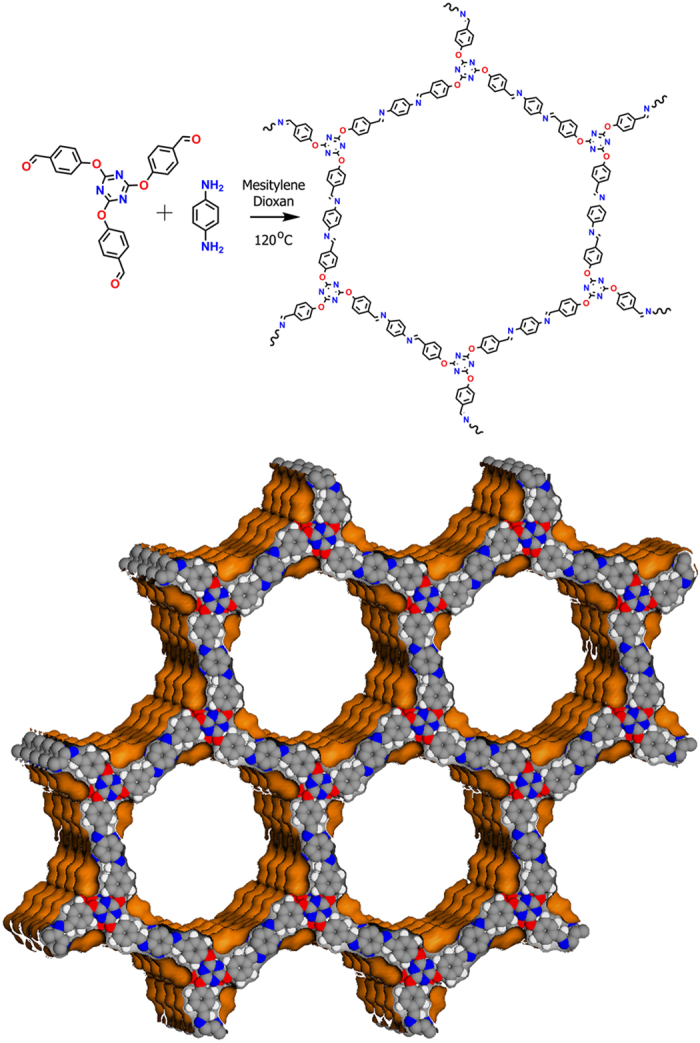
Top: Schematic representation of the monomeric units and the final COF structure. Bottom: Shows the Connolly representation of the three dimensional framework formed by the stacking of the hexagonal layers in a P6/mcc setting. The one-dimensional channels run along the c-axis (29 Å).

**Figure 2:**
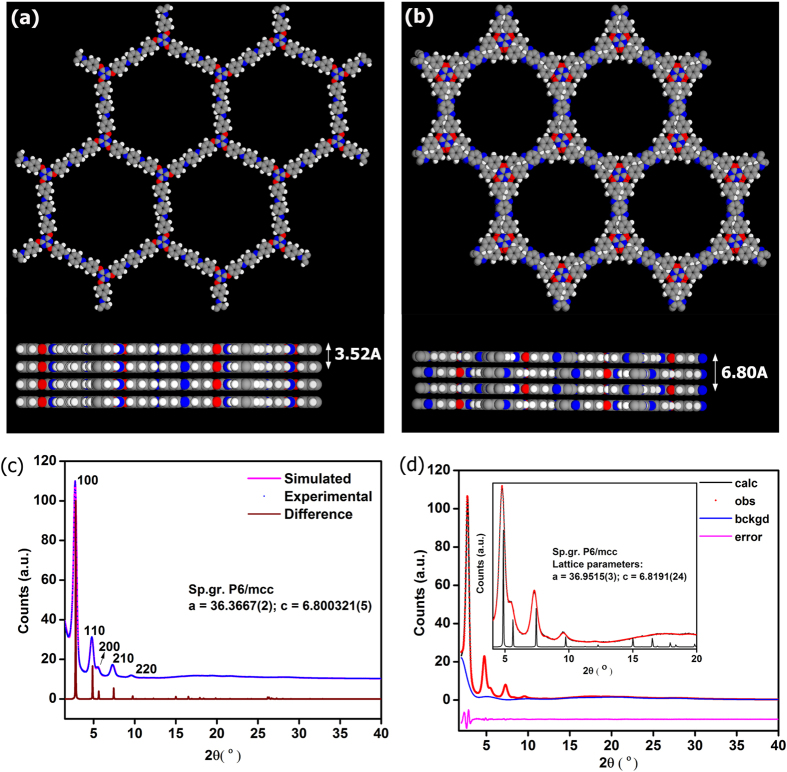
(**a**) Hexagonal honeycomb layers of trzn-COF in P6/m setting, with large 1-D pores (34 Å) and the AA… type stacking with an interlayer separation of 3.52 Å. (**b**) Circular channels (29 Å) created by the stacking of hexagonal layers in P6/mcc setting. The stacking is of *ABAB*… arrangement with a C-axis length of 6.80 Å. (**c**) A Pawley fit in P6/mcc setting. The statistics associated were good and comparable to those obtained for P6/m setting. (**d**) A Le bail fit for the COF in P6/mcc (χ^2^ = 3.512; R_p_ = 0.0264; wR_p_ = 0.0336). Inset shows the goodness of the fit with markers for P6/mcc. It also shows the lack of many peaks in the 2θ range of 12-16°, where P6/m would have more peaks than P6/mcc. Color code: O- red; N- blue; C- grey and H- white.

Presence of highly flexible appendages in the building units posed significant challenges during structure solution, nevertheless, a structure consistent with the experimentally observed powder pattern has been obtained using combination of GSAS and crystal building simulations carried out using *Accelrys* program. The monomer employed in the trzn-COF synthesis is among one of the longest and flexible ones reported, and would not be expected to crystallize in a high symmetry structure (Fig. 1). Most of the COFs made of 3-connecting node and ditopic linker have been reported with hexagonal layered structure crystallized in P6/m space group and very few in higher symmetries (P6/mmc or P6/mmm)^15^,^54^,^55^. For the trzn-COF, a simple hexagonal layers with eclipsed arrangement in P6/m would pose an extreme demand on the structure owing to its long linkers and highly flexible functionalities. Foreseeing this, we carried out an extensive crystallographic screening using a combination of Pawley and Le bail refinements (Fig. 2).

The powder XRD was indexed using a X-Cell program. Then, following a profile fit, the space groups search was carried out using Pawley routine. The space groups P6/m and P6/mcc were identified with well acceptable FOM (>20), of which the P6/mcc had the highest value. The powder pattern was analyzed, in this setting, using Le bail fit. An excellent intensity agreement and fit was obtained from Le bail for both P6/mcc and P6/m (Fig. 2d) (P6/mcc: χ^2^ = 3.512; R_p_ = 0.0264; wR_p_ = 0.0336) and P6/m: χ^2^ = 3.406, R_p_ = 0.0230 ; wR_p_ = 0.0320,). When a Pawley fit was carried out in both space group settings, they both had good statistics (P6/m: R_p_ = 2.59, wR_p_ = 3.51, P6/mcc: R_p_ = 2.86, wR_p_ = 4.01) (Fig. 2c). Other space groups such as P3, P-3 produced a structure consistent with the experimental powder pattern, but were not considered as they merely represent the lower symmetry sub-groups.

It was difficult to differentiate between the two space groups, P6/m and P6/mcc, based on relative intensities. The major differentiating region in the pxrd was between 2θ of 12° to 16°, a comparison of the simulated patterns in this region with the experimental one showed relatively better fit for P6/mcc (Figure s1). Finally, when the energy associated with P6/m (−174 Ha) and P6/mcc (−348 Ha) were estimated from the tight binding DFT routine (DFT-TB), it could be observed that the P6/mcc was favorable. This could be due to the higher packing efficiency in the latter space group. Thus, we propose the correct structure as being P6/mcc with an *ABAB…* stacking, giving an interlayer separation of 6.8 Å.

The framework of trzn-COF is formed by the Schiff base linkages (C=N) between the triazine core based trialdehyde and P-phenylenediamine. The presence of ether linkages could provide considerable stability in water or atmospheric moisture. In fact the vapor sorption studies carried out on the trzn-COF indicates lack of any strong interaction with water. Repeated water sorptions show no loss in uptakes suggesting the water stability of the compound and this is consistent with the stability assessed from PXRD done on a sample that was soaked in water at room temperature for 24 hrs (Figure s3). When investigated from PXRD studies, the as-made form of trzn-COF did not show any pore shrinkage even upon evacuation (no shift is observed in the *100* peak, 2θ = 2.69). Pawley refinements carried out using the *Materials Studio* (*version 4.4)* gave excellent fit and most of the low angle peaks could be assigned. The *002* peak, corresponding to the interlayer spacing of 6.8 Å is observed at 2θ = 26.2° (Figure s1). trzn-COF crystallizing in such high symmetry space groups certainly defies intuition and could prompt revisiting of several highly functionalized monomeric units for making COFs, which could have been ruled out by many.

**Figure 3:**
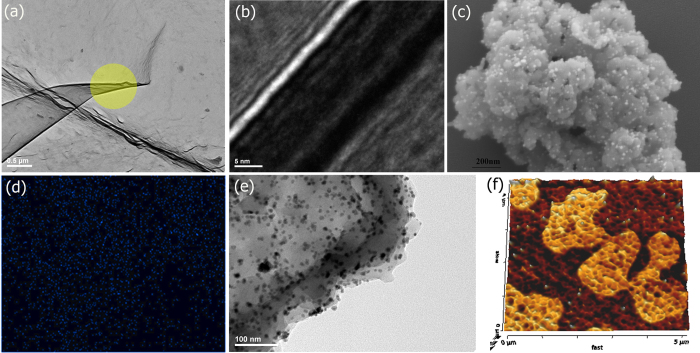
(**a**) HR-TEM image of the as-synthesized trzn-COF showing the transparent layers formed by the COF and a fold can be seen at the region where the layer wraps-up. The yellow circled region has been zoomed in the adjacent figure. (**b**) A zoom-in using HRTEM on the folds of the COF layers (yellow circle, Fig. 3a) showing the grid like appearance within and the flaky appearance between the trzn-COF layers (**c**) Field emission SEM of Pd-trzn-COF showing the uniform morphology of the COF, and the homogeneous loading of Pd^0^. (**d**) the elemental mapping of Pd^0^ showing its uniform distribution across the COF surface. (**e**) a high resolution TEM image of the Pd-trzn-COF showing the presence of small nanoparticles of Pd^0^ (<5 to 20 nm) on the surface of the COF. (f) A 3D AFM image showing the ordered patterns formed by the trzn-COF (relative heights, dark brown: 0nm; light brown = 20nm).

A ^13^C-SSNMR contained peaks in the range of 120 to 190 ppm, which could be assigned to seven different carbons (supporting information). Thermogravimetric analyses showed the trzn-COF is stable up to 350 °C (Figure s2). The Field Emission-SEM (FE-SEM) of the trzn-COF shows a high degree of homogeneity with flakes clustered to form spherical entities (Figure s4). High Resolution-TEM (HR-TEM) showed the COF as thin transparent and smooth layers (Figure s5) and some of the layers were folded at the ends (Fig. 3a). A cross-sectional view along the folds of the COF layers using HR-TEM showed a wafer like appearance with regular patterns on the surface and stacking could be seen along the orthogonal direction (Fig. 3b).

### Preparation and characterization of Pd^0^-trzn-COF catalyst

Pd^0^ nanoparticles are loaded by stirring trzn-COF in an ethanolic solution of Pd(OAc)_2_. Interestingly the reduction of Pd^2+^ and the formation and loading of Pd^0^ nanoparticles into the COF happens in this single step. In many cases a separate reduction step has been carried out using strong reducing agents such as NaBH_4_^38^, LiAlH_4_ or hydrazine or amines^25^. Except in a few cases, these reducing agents may hydrolyze the framework^38^. We believe the richness of basic nitrogen in the COF has a role to play in this reduction. EDAX and elemental analyses indicated a 18–20% Pd loading (Figure s7).

**Figure 4:**
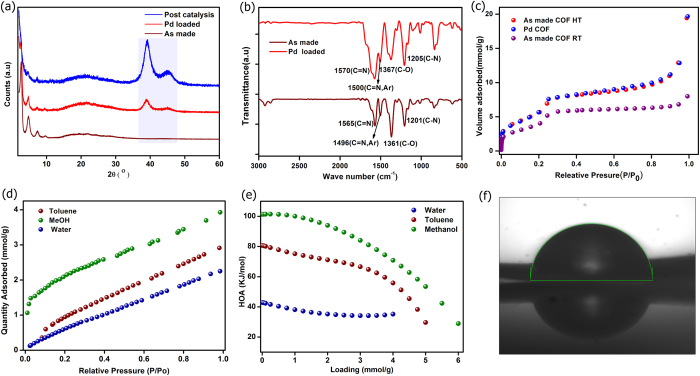
(**a**) Comparison of the PXRD patterns of the as-synthesized trzn-COF, Pd-trzn-COF and the post catalysis Pd-trzn-COF indicating considerable stability to nanoparticle loading and catalysis. (**b**) IR spectra showing the presence of expected functionalities and a good match between the catalyst and the support (**c**) Nitrogen adsorption isotherm of trzn-COF and Pd-trzn-COF carried out at 77K. It has micro- and mesoporous character. The low temperature synthesized phase show about 35% lower porosity than the HT one. (**d**) Vapor sorption isotherms of trzn-COF showing least interaction towards water and a more abrupt uptake of methanol at low P/P_o_ region. (**e**) Heats of adsorption data showing the interaction being the strongest for methanol. (**f**) Contact anlge measurement showing the angle made by a drop of water on a powder surface of the COF (90 ± 2°).

The catalyst has been characterized unambiguously through PXRD, TGA, IR, XPS, FE-SEM, EDAX-elemental mapping, TEM and SSNMR (Fig. 4, supporting information). PXRD of Pd-trzn-COF matches quite well with that of the as-synthesized one (Fig. 4a). TGA of the Pd-trzn-COF shows a continuous and gradual weight loss above 140 °C concurrent with those observed in the literature (Figure s2)^25^,^38^. When the TGAs corresponding to Pd-trzn-COF treated under vacuum at different temperatures (120, 150, 180 and 200 °C) were compared, it could be seen that the occluded solvents and any adsorbed moisture could be removed without any structural degradation at these temperatures (Figure s2).

FE-SEM of the Pd-trzn-COF shows the presence of highly dispersed loading and they seem to have the particles with sizes ranging from <5 to 20 nm (Fig. 3c). This could mean the particles can be dispersed both within the pores of the COF as well as the surfaces. Being able to image Pd on COF with such clarity from a FE-SEM is quite remarkable. Pd^0^ is known to form smaller nanoparticles compared to Pd^2+^^46^. A HR image from the FE-SEM shows presence of patterned surfaces (Figure s4) and the Pd nanoparticles are uniformly distributed over them, this is further confirmed by the elemental mapping of the Pd exactly at the same region (Fig. 3d). The HR-TEM further confirmed the even distribution of Pd nanoparticles with a general size range of 5–20 nm and few with sizes <5 nm could also be seen (Fig. 3e). AFM images of the dilute drop-casted samples showed a granular micro pattern (Fig. 3f and s6) with nanometer-scale pores and some macropores.

The IR of both trzn-COF and Pd-trzn-COF match very well and show typical bands corresponding to triazine core and no appreciable carbonyl stretching bands were observed indicating the lack of any Pd(OAc)_2_ loading (Fig. 4b). The alcohol based condition and given the presence of the basic triazine units, it is unlikely that PdO could form. Furthermore, the XPS analysis shows presence of only Pd^0^ (Binding energies: Pd-3d_5/2_ = 334.7 and Pd-3d_3/2_ = 339.9) in the Pd-trzn-COF (Figure s8), thus, the employed loading conditions favor complete reduction of Pd^2+^. Importantly, the material was subjected to 130 °C in DMF during the catalysis operations and a mass balance suggested no leaching of Pd. To further substantiate, we isolated the supernatant solutions and evaporated them to dryness. When this extract was analyzed under EDAX, no trace of Pd was observed.

Surface area and pore size distribution of trzn-COF and Pd-trzn-COF were determined using N_2_ adsorption isotherm measured at 77K (Fig. 4c). The DFT fit to the isotherm showed presence of micro and mesopores, with micropores being in lower concentration (Figure s9). Anticipating the probability of the bimodal pore character occurring from impure synthesis, synthesis were repeated and several batches were screened for N_2_ isotherms. Across all batches similar uptakes and isotherm profiles could be obtained. Considering the very less dense powder character of the material and based on the microscopy images, we anticipated some inter-particle spaces that are in the micro-mesoporous regime. For this reason we prepared this sample differently using methods like mechanical grinding and/or sonication and carried out the N_2_ adsorptions. All such studies gave a same isotherm profile with an isotherm showing microporous behavior at low P/P_0_ and with no decrease in uptake. The as-synthesized form of trzn-COF has a BET surface area of 408.5 m^2^/g, while the Pd-trzn-COF possessed a BET surface area of 404 m^2^/g. Considering this unusual case, wherein there is not much of a loss of surface area upon Pd loading, a non-model dependent fit, Barrett-Joyner-Halenda (BJH), was also used to obtain the pore size. The as-synthesized form had mesopore of 23 Å, while the Pd loaded form had 19 Å pores. While the Dubinin-Radushkevich (DR) model gave a pore volume of 0.21cc/g and a surface area of 563 m^2^/g.

It is quite unusual trzn-COF did not lose any N_2_ uptake on Pd^0^ loading unlike other Pd^2+^ loaded COFs^25^,^38^. This could suggest the presence of only Pd^0^ in our COF as it occupies much lesser pore space than Pd(OAc)_2_ and even while sitting on the surface of the COF, owing to its smaller size would be expected to create less impediment to the pore access. A Non Localized Density Functional Theory (NLDFT on carbon) fit carried out to estimate the pore size distribution showed: though the total surface area did not decrease between the as-made and the Pd loaded phase, the pore volumes were not hugely different. While trzn-COF showed pore sizes of 12.7 Å and 27 Å, the Pd-trzn-COF showed pore sizes of 18 Å and 27 Å with exactly same ratio. To further expand on the origin of micropores in this material, we realized that such hierarchical micro-mesopores have been observed in other carbonaceous material and different annealing temperatures have been used to distinguish between point defects giving rise to micropores, while stacking faults generating mesopores^56^. While the latter can be removed by annealing, the former has been shown to be inherent to and persistent in the material. When a similar analysis was carried out on our material, by heat treating the material at 120, 150, 180 and 200 °C, it did not result in any change in the profiles or uptakes of the isotherms. In conclusion, if a model independent BJH is used the material is found to have mesopores (23 Å) which is in reasonable agreement with the pore size estimated from the described structure. And this decreases to 19 Å on Pd loading.

Several pyridyl or amine based complexes and extended supports have been shown to interact with Pd^2+^ via both p and d bonding and hydrogen bond type interactions^57^,^58^. But, interactions of Pd^0^ with such groups are not much explored. Hence to better understand the surface of the trzn-COF and its favorability to interacting with Pd nanoparticles, a series of vapor sorption measurements have been carried out and heats of adsorption data has been analyzed. Water showed a near-linear isotherm with no appreciable concavity or convexity, indicating lack of any strong interaction with the surface. While the non-polar toluene showed much more abrupt uptake and concavity towards the pressure axis indicating stronger interactions, while methanol has a most abrupt uptake at low P/P_0_ compared to water or toluene. Which is indication of stronger interaction particularly at lower pressures, which is reflected in a very strong heat of adsorption for MeOH at the zero-loading (Fig. 4d). This suggests the surface is considerably polar and hydrophobic, thus showing an *‘amphiphilic’* nature. And, in spite of having ether lining and relatively non-polar groups on the walls of the pore, trzn-COF did not show much selectivity between methanol and toluene. This is further confirmed by the heats of adsorptions calculated using a virial model (Fig. 4e). Water had the least value (40 kJ/mol), which is just above the vaporization point of water^59^, suggesting very weak interactions, while toluene showed an HOA of 80 kJ/mol and methanol seems to interact the best with the surface with a value of 100 kJ/mol. These observations were in accord with the contact angle of 90 ± 2° measured for trzn-COF (Fig. 4f).

**Figure 5:**
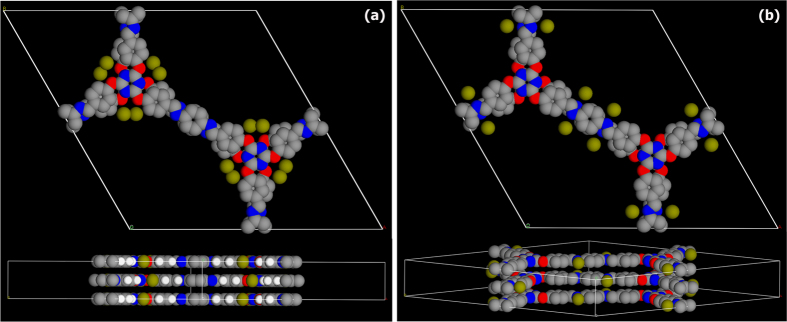
A DMol^3^ (DFT-D corrected) energy and geometry optimization of the Pd-COF interactions. Several starting models with different Pd position were attempted and it yielded two low energy configurations. (**a**) In the minimized geometry, the Pd atoms resided in the small clefts formed around the triazine core lined by the ether and the N and C of the triazine rings. A b-axis view showed that they were lined well with the layer. (**b**) Another minimized configuration included the interaction of the Pd atoms with the nitrogens of the Schiff bond. It can be seen that the Pd atoms align with the N atoms. In both cases the unit cell was retained and framework atoms were frozen in P6/mcc configuration. Color code: Pd- olive green; O- red; N- blue; C- grey and H- white.

To further verify the favorable sites in trzn-COF for interactions with Pd^0^ and the effectiveness of triazine cores to serve as interaction sites with Pd, modeling studies have been carried out. The interactions were modeled using a DMOL^3^ program with DFT-D corrections (*Accelrys*). In P6/mcc unit cell, different starting configurations were generated by distributing the Pd atoms in specific sites (closer to the framework). When each such configuration was allowed to geometry optimize, it was found that the most energetically favorable sites were the small cleft formed around the triazine ring and the ether bonds (320 kcal/mol) as well as sites proximal to the nitrogens of the Schiff bonds (319 kcal/mol, Fig. 5). Energy associated with Pd located in other sites (example: in the middle of the pore, closer to the aromatic backbone *etc*.) were much higher. Yet, a detailed study would be required for more tangible insights.

## Catalysis by Pd-trzn-COF, multi-fold Heck couplings and C-C bond formation reactions

In order to evaluate the catalytic activity of Pd-trzn-COF towards multiple-site/multi-fold Heck couplings and C-C bond formations using non-boronic acid substrates several reactions were carried out (Tables s1, s2 and s3). Most common Heck and Suzuki catalysts involve Pd^2^^+^ and the substrate is a boronic acid^44^,^60^. Non-boronic acid based C-C coupling have been done via Ullmann reactions^60^ (Common catalyst: CuX, X = halide, organic groups, sulfate etc) using a variety of substrates and metal salts (Cu, Ni, Zn, Fe) as catalysts. In many cases they employ high temperatures (>200 °C)^61^, or high catalyst concentrations when carried out at low temperatures (>5mol%)^60^. Though the Ullmann type reactions can operate on expansive range of substrates, the high and consistent yield obtainable from boronic acid based Suzuki couplings make them the most attractive route irrespective of their expensive ingredients. Given these contrasting features, making novel heterogeneous catalyst capable of working in very dilute concentrations across a variety of substrates under ambient conditions is desired. Some of the lowest catalyst concentrations employed during Suzuki couplings have been in the case of Pd doped quaternary systems with perovskite structures (TON=27000)^62^. The catalyst concentration employed while using Pd-trzn-COF are comparable to the ones observed in some of the most optimized (optimized over 20years) homogeneous Heck reactions^63^.

One of the major challenges in Heck type reactions is in achieving multi-fold substitution to synthesize multi-functionalized final products in pure form with high yields^47^,^64^. Table 1 lists the multi-fold Heck coupling reactions carried out. Most of the compounds have been isolated in excellent yield (80% to 95%, isolated yield) and with high TON (Table 1). Reports on many of these reactions carried out using a variety of catalysts are prevalent, but they involve multiple steps and there is a strong drive to achieve these reactions via a one-pot route^65^,^66^. Of particular mention is the products, biphenyl-4,4’-dicarboxylic acid (bpdc), 3,3’,5,5’-biphenyltetracarboxylic acid, 1,3,6,8-tetra(styryl)pyrene and a few other shown in Table s2. The bpdc is a commercially sold and widely used organic ligand in the formation of MOFs and other advanced materials. Another product, the styryl-pyrene could serve as an excellent monomer for developing functional polymers. To demonstrate the activity of the catalyst for heterogeneous couplings in aqueous media, we carried out Suzuki-Miyaura coupling using boronic acid substrates and we could obtain expected products in >99% yield (Table s3). The catalysts did not show any appreciable loss of activity for all three categories of reactions when tested over 4 cycles.(Figure s17). Encouraged by the possibility of being able to disperse the organic COF into porous polymers to develop membranes, we dispersed trzn-COF and Pd-trzn-COF into Poly(methyl methacrylate) (PMMA) and made membranes by spin coating techniques. Via this method a loading of up to 50% could be achieved. The resulting composite can be made into membranes of different shape and size (For further details see supporting information).

**Figure 6:**
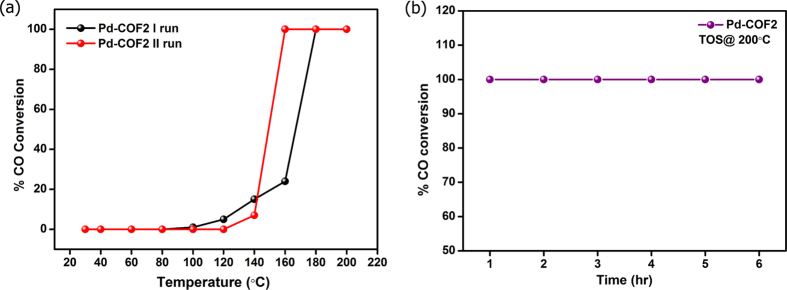
(**a**) The CO conversion plot for Pd-trzn-COF for two cycles of activity testing (**b**) Time of stream of Pd-trzn-COF at full conversion.

Oxidation of CO represents a prototype reaction to demonstrate gas phase catalysis by metal nanoparticles particularly in supported form^52^,^67^. To our knowledge, COF supports have not been investigated for this application. CO oxidation activity test was carried out to assess the ability of Pd-trzn-COF catalyst for catalysing gas phase reaction. The Fig. 6 shows the CO conversion plot for the Pd-trzn-COF catalyst during the two cycles of activity testing. During the first run the catalyst showed an onset temperature of 100 °C with a full conversion at 180 °C.The TOS data clearly showed that there was no loss in activity over extended period of time. The second cycle of the catalyst again proved their stability, where it showed a light off temperature at 140 °C and drastic increase in activity to full conversion at 160 °C. This results coincide well with that of the performance of Pd/Al_2_O_3_ catalyst^45^.The CO oxidation activity of Pd nanoparticles with size less than 8 nm and on different supports is well documented in literature^68^,^69^,^70^. The enhanced activity of trzn-COF supported Pd catalyst can be attributed to the lack of metal leaching and catalyst poisoning^71^. The spent catalyst showed the catalyst to be sinter resistant (Figure s18).The other novelty of the synthesis strategy is that it is surfactant free synthesis which reduces the number of activation/pre treatment steps. This study implies that COFs can mimic the performance of true industrial catalyst working under harsh conditions without any appreciable loss of activity. The Pd-trzn-COF did not show any substantial interaction with H_2_ at 77 K or 303 K and O_2_ at 273 K or 303 K, as observed from respective adsorption measurements. Thus indicating an apparent stronger chemical interaction with CO over these gases (see supporting information for details).

## Conclusion

The COF presented here is made up of long and flexible appendages, yet, crystallizes into a well-ordered thermodynamically stabilized structure (hexagonal, P6/mcc). It has well-defined micro (13 Å) and mesopores (27 Å). Presence of Palladium-specific interaction sites with basic character, triazine and imine groups, favors a facile, one-pot loading of Pd nanoparticles in them via a room temperature reduction. The homogeneity of the catalyst loadings have been shown using a series of microscopy methods. The amphiphilic character of the COF surface ensuring water stability, but providing optimal polarity comparable to homogeneous solvents has been established via vapor sorptions. The sites proximal to triazine and ether links seem to be favorable for Pd interactions, while the Schiff bonds constitute another interaction site capable of providing nearly equal energy stabilization. The work should definitely prompt considering several other highly flexible and functionalizable groups in making such high symmetry structures. And the range of catalysis with the exceptional activity and stability can be attributed wholly to the fine dispersion of Pd nanoparticles (<5–20 nm) on to the COF surface. The study promises the potential of noble metal-COF composites in developing many interesting organic molecules via multi-fold Heck coupling reactions and non-boronic acid supported C-C bond formations.

## Additional Information

**How to cite this article**: Mullangi, D. *et al*. Pd loaded amphiphilic COF as catalyst for multi-fold Heck reactions, C-C couplings and CO oxidation. *Sci. Rep*. **5**, 10876; doi: 10.1038/srep10876 (2015).

## Supplementary Material

Supplementary Information

## Figures and Tables

**Table 1:**
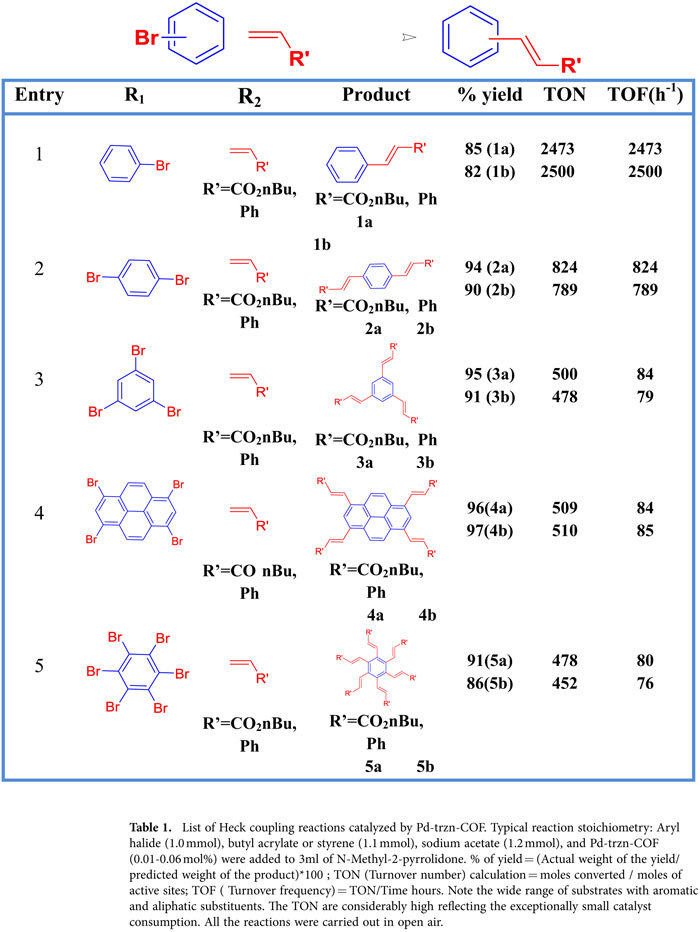
List of Heck coupling reactions catalyzed by Pd-trzn-COF.
