# Supplementary material for: Pd loaded amphiphilic COF as catalyst for multi-fold Heck reactions, C-C couplings and CO oxidation
Source: Sci Rep. 2015 Jun 9;5:10876. doi: 10.1038/srep10876 (PMC4603777; doi:10.1038/srep10876)

*Supporting Information*

# Pd loaded amphiphilic COF as catalyst for multi-fold Heck reactions, C-C couplings and CO oxidation

Authors: Dinesh Mullangi[1], Shyamapada Nandi[1], Sorout Shalini[1], Sheshadri Sreedhala[2], Chathakudath P. Vinod[2] and RamanathanVaidhyanathan[1*]

Affiliations: [1]Department of Chemistry, Indian Institute of Science Education and Research, Pune, India.
[2]Department of Chemistry, National Chemical Laboratories, Pune, India.

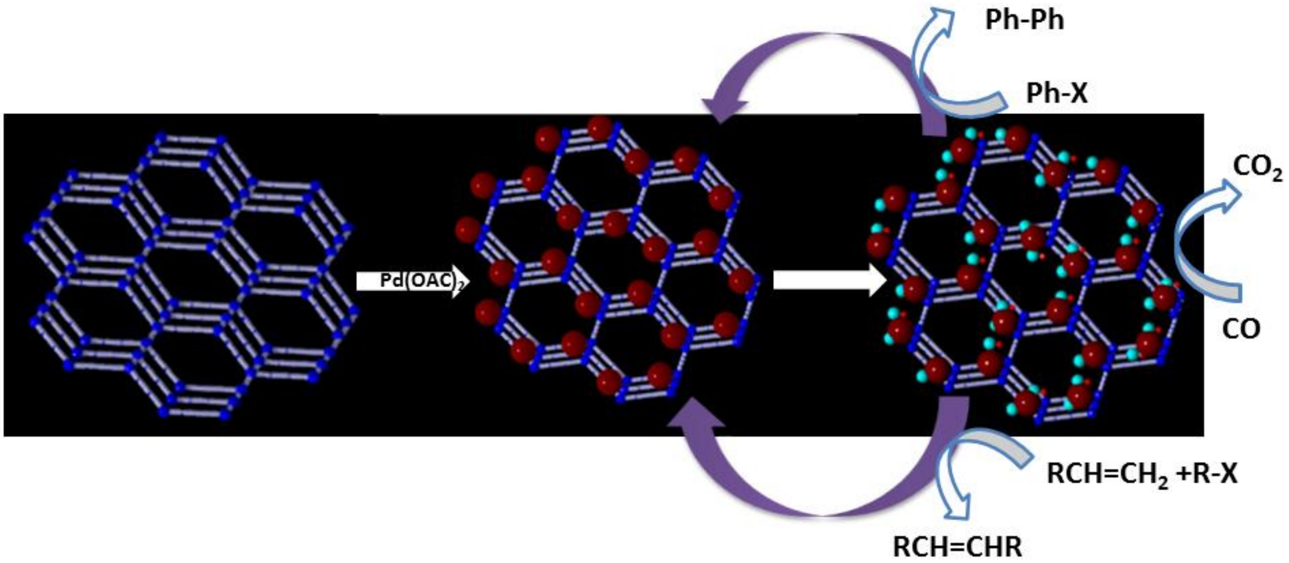

## *List of contents*


1. ***Materials and methods (Page 3)***
2. ***Analytical characterizations (Page 7)***
3. ***Microscopy: SEM analysis, HRTEM analysis, AFM Studies (Page 11)***
4. ***EDAX analysis (Page 14)***
5. ***XPS Spectra (Page 16)***
6. ***Adsorption data: $N_2$ sorption Measurements, Solvent sorption Measurements (Page 17)***
7. ***Catalysis: Heck couplings, C-C couplings, Suzuki-Miyaura couplings and CO oxidation (Page 23)***
8. ***Composites: trzn-COF-PMMA and Pd-trzn-COF-PMMA (Page 26)***
9. ***Appendix: NMR Spectral data for catalysis, Single crystal structures of products( Page 28)***

# 1. Materials and methods

All the organic chemicals were purchased from Sigma Aldrich and were used without any further purification.

**Synthesis of monomers**

2,4,6-Tris(p-formylphenoxy)-l,3,5-triazine was synthesized according to the reported procedure.(S1) and p-phenylenediamine was purchased from Sigma Aldrich.

(s1). *Deborah C. T and Tomikazu S; J. Org. Chem. 1994,59, 679-681.*

**Synthesis of trzn-COF**

2,4,6-Tris(p-formylphenoxy)-l,3,5-triazine (TRIPOD) (100 mg, 0.23mmol) 1,4-diaminobenzene (48 mg, 0.46mmol) were dissolved in 1,4-dioxane (5.0 mL) in a Pyrex tube, to this mixture mesitylene (5.0mL) was added and the contents were homogenized by stirring. Following this, about 0.5 mL of aqueous acetic acid (3M) was added. Then the Pyrex tube was flash frozen in a liquid nitrogen bath, the free space was evacuated and the tube was closed under a blanket of nitrogen. The tube was placed in an oven at 120 ºC for 3 days. A brown solid was obtained, which was washed with DMF , dioxane , acetone and THF. The yield was about 110mg (87%). Activation of the sample for gas adsorption was done by soaking the sample in THF for 3days with three time replenishment of the solvent. (CHN Analysis: Obsd. C = 66.9; H = 3.74; N = 17.38. Calc. 70.9; H = 5.41; N = 15.04, (Note: the CHN values have been calculated using a COF made with a TRIPOD monomeric unit to phenylenediamine ratio of 2:3. This is the ratio employed in the synthesis).

**Synthesis of Pd-trzn-COF**

Palladium acetate (36 mg,) was dissolved in 10 mL of Acetone and the trzn-COF (100 mg) was dispersed in 20mL of ethanol. The COF dispersion was added to the Pd acetate solution using a dropping funnel or a syringe needle over 30mins. The mixture was stirred for 24 hrs at room temperature. The resulting black solid was filtered and washed with acetone, ethanol and THF dried over vacuum for 12hrs. (CHN Analysis: Obsd. C = 43.3; H = 2.6; N = 11.6. Calc. (**Formula: trzn-COF.(Pd)$_{3.6}$**: 42.3; H = 3.06; N = 9.1)

**Room temperature synthesis of trzn-COF**

2,4,6-Tris(p-formylphenoxy)-l,3,5-triazine (TRIPOD) (100 mg, 0.23mm) 1,4-diaminobenzene (48 mg,0.46mmol) were dissolved in 1,4-dioxane (5.0 mL), to this mesitylene (5.0mL) was added and stirred until it became homogeneous, to this solution 0.5 mL of aqueous acetic acid (3 M solution) was added. Immediately an orange-yellow precipitate started to appear. Following this, the reaction mixture was stirred for additional 5 mins. Contents were left standing at RT for 3 days. The precipitate, a light brown solid, was filtered and washed with copious amounts of 1,4-dioxane, MeOH DMF, THF and Acetone. The procedure

yielded 106mg of the trzn-COF (~83%). CHN analysis: C = 66.48; H = 3.73; N = 17.58. Calc. 70.9; H = 5.41; N = 15.04. This sample gave lower porosity compared to the sample obtained from 120°C synthesis.

Based on porosity as the major aspect, we gave more priority to higher temperature synthesised COF and all preliminary studies were done for RT-COF expect catalytic applications. PXRD indicated the room temperature sample to be more crystalline compared to the 120°C synthesis.

**General Procedure for Heck reaction**

Aryl halide (1.0 mmol), butyl acrylate or styrene (1.1 mmol), sodium acetate (1.2 mmol), and Pd-trzn-COF (1mg) were added to 3ml of N-Methyl-2-pyrrolidone (NMP). The reaction mixture was stirred at 120°C for 1hr in open air, whereas in the case of di to hexa bromination, the reaction was carried out over 6-10 hrs and 3 to 5mg of catalyst was used. At the end of the reaction (as monitored from TLC), the reaction mixture was poured in to water and extracted with DCM. DCM was evaporated under reduced pressure. Products were purified by column chromatography and characterized by using NMR spectroscopy.

For recyclability tests, a reaction involving p-nitrobromobenzene (101 mg, 0.5 mmol), n-butylacrylate (74 mg, 0.575 mol), sodium acetate (51mg, 0.625 mmol), and Pd-trzn-COF (0.5 mg) in 3mLof NMP was considered. After each cycle, the reaction mixture was centrifuged and the catalyst was recovered, for the next cycle it was used directly without any further treatment.

**General Procedure for Ulmann type (C-C) coupling**

Aryl halide (0.5mmol), Potassium Carbonate (0.6mmol), and Pd-trzn-COF (2mg,) were added to 3ml of DMF. The reaction mixture was stirred at 120°C for 6hrs in open atmosphere. After completion of reaction (as monitored by TLC), the reaction mixture was poured in to water and extracted with DCM. DCM was evaporated under reduced pressure. Products were purified by column chromatography and characterized by using NMR spectroscopy and some of them were isolated as single crystals and characterized using SCXRD.

**Suzuki Coupling**

**Reaction conditions:**

Aryl halide (1.0 mmol), phenylboronic acid (1.1 mmol), NaOH (1.2 mmol), Tetra-n-butylammonium bromide (TBAB) (1.2 equiv), 1mg of Pd- trzn-COF were added to 3mL of water. The reaction mixture was stirred at 65°C for 4hrs in open air. After completion of reaction, the reaction mixture was poured in to water and extracted with DCM. DCM was evaporated under reduced pressure. Products were purified by column chromatography and characterized by using NMR spectroscopy and in some cases using SCXRD.

**$CO$ to $CO_2$ Oxidation experimental conditions:**

The catalytic activity of supported Pd- trzn-COF catalyst for CO oxidation was measured in a fixed bed reactor under atmospheric pressure using 100 mg pelletized catalyst. The temperature was ramped between 30°C to 300°C at 2°/min ramping rate. The total flow rate was 50 ml/min with a ratio of (1:5:19 CO: $O_2$: $N_2$). The calculated GHSV was 30000 $cm^3$/ gcat/ hr. The temperature of tubular furnace where the reactor was mounted was controlled by Radix6400 temperature controller and the catalyst bed temperature was measured by a K-type thermocouple. The effluent gases were analysed online by gas chromatograph equipped with online gas sampling valve and a TCD detector. The activity was examined by looking at the CO conversion.

$$\text{CO conversion (\%)} = \frac{CO_{initial} - CO_T}{CO_{initial}} \text{ X } 100,$$

Where $CO_T$ is amount of consumed at particular temperature T.

**COF-PMMA composite preparation:**

A SPEKTROSPIN spin coater was used for making the films of trzn-COF/PMMA and Pd-trzn-COF/PMMA composites on glass substrate. Before spin coating the substrate was cleaned with soap and distilled water. It was then soaked in IPA and acetone for half an hour.

In a typical process, a suspension of 50 mg trzn-COF/Pd-trzn-COF and 50 mg PMMA (50% weight loading of COF in PMMA) in 5 ml THF was heated at 65°C for 12 hours with vigorous stirring. The solution, while hot, was dropped using a dropper on to the substrate maintained at ~60$^oC$, in a quantity just enough to cover it. It was spun at room temperature at 500 rpm for 60s, 1000 rpm for 60 s, 1500rpm for 60s and 2000 rpm for 60 s. This was repeated as long as the desired thickness of film was obtained. To remove the extra solvent, the film was dried in open air for 30 minutes and then placed in a UV curer for 30 minutes after spin coating. To peel off the film from the glass substrate, it was soaked in distilled water for 2 minutes. The obtained film was again dried in an oven for half an hour.

**trzn-COF monomer structure and characterization:**

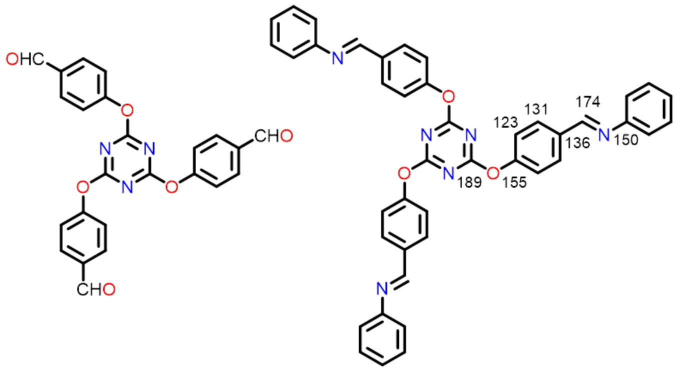


**Tri aldehyde Solution NMR data:**

$^{1}$H NMR (400 MHz, CDCl3) δ 10.00 (s, 3H), 7.93 (d, J = 8.4 Hz, 6H), 7.33 (d, J = 8.4 Hz, 6H).

$^{13}$C NMR (101 MHz, CDCl3) δ 190.32, 172.94, 155.40, 134.17, 131.01, 121.91, 77.16.

COF2 $^{13}$C-SSNMR data

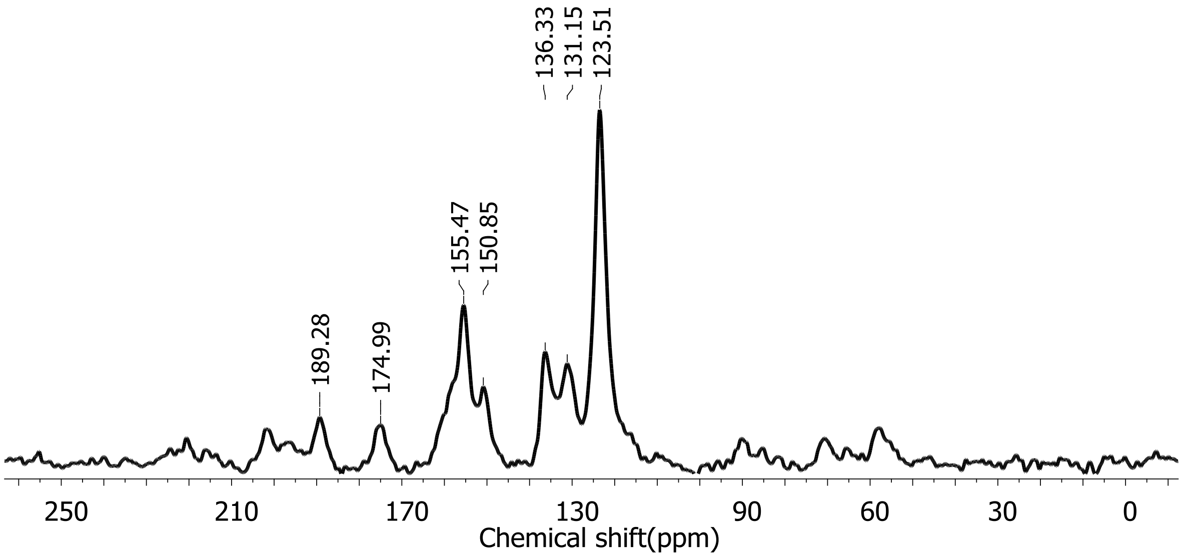


Solid state $^{13}$CNMR for trzn-COF as made form. Bottom is a schematic of the monomer showing the peak assignments.

## 2. Analytical characterizations

Powder X-ray diffraction:

Powder XRDs were carried out using a Rigaku Miniflex-600 instrument and processed using PDXL software and for some cases Bruker Discover.

Thermogravimetric Analysis:

Thermogravimetry was carried out on NETSZCH TGA-DSC system. The routine TGAs were done under $N_2$ gas flow (20ml/min) (purge + protective) and samples were heated from RT to 500ºC at 2K/min.

FEI (model Tecnai F30) high resolution transmission electron microscope (HRTEM) equipped with field emission source operating at 300 KeV was used.

X-Ray photoelectron spectroscopic (XPS) measurements were carried out on a VG Micro Tech ESCA 3000 instrument at a pressure of >1 x $10^{-9}$ Torr (pass energy of 50 eV, electron take-off angle of 60°, and overall resolution was 0.1 eV).

Ultra Plus Field Emission Scanning Electron Microscope with integral charge compensator and embedded EsB and AsB detectors. Oxford X-max instruments 80mm$^2$. (Carl Zeiss NTS, Gmbh), Imagin conditions: 2kV, WD=2mm, 200kX, Inlens detector.

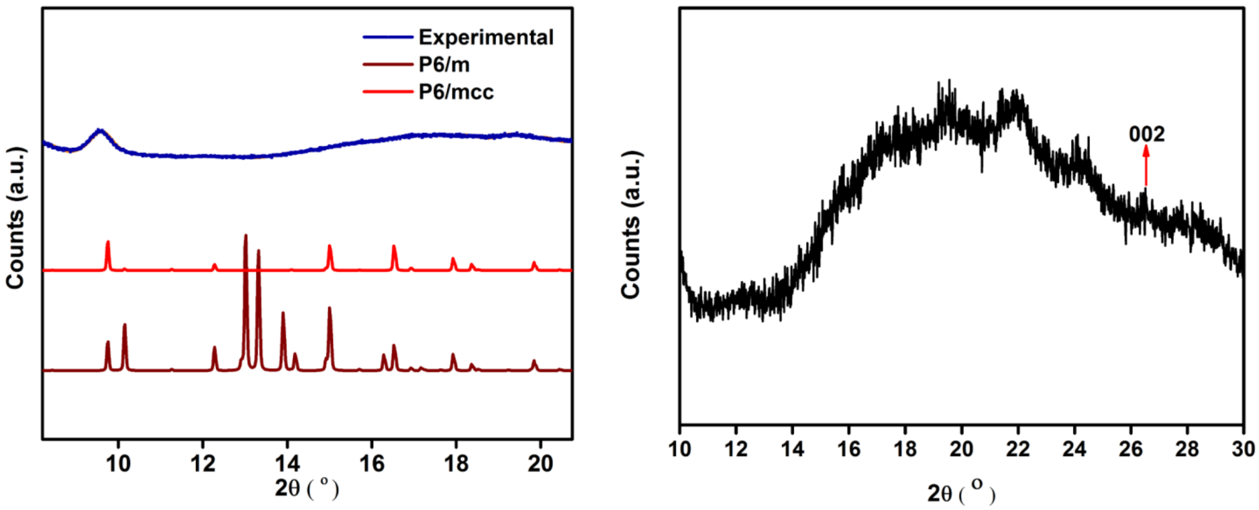


**Figure s1.** Left: PXRD of the as-synthesized form of trzn-COF being compared to the simulated patterns of P6/m and P6/mcc, showing the lack of peaks expected for the P6/m in the 12° to 16° 2-theta region. Thus indicating a better fit to P6/mcc. Right: Shows the 002 peak at 2θ= 26.2° corresponding to the interlayer spacing of 6.81Å in the P6/mcc setting.

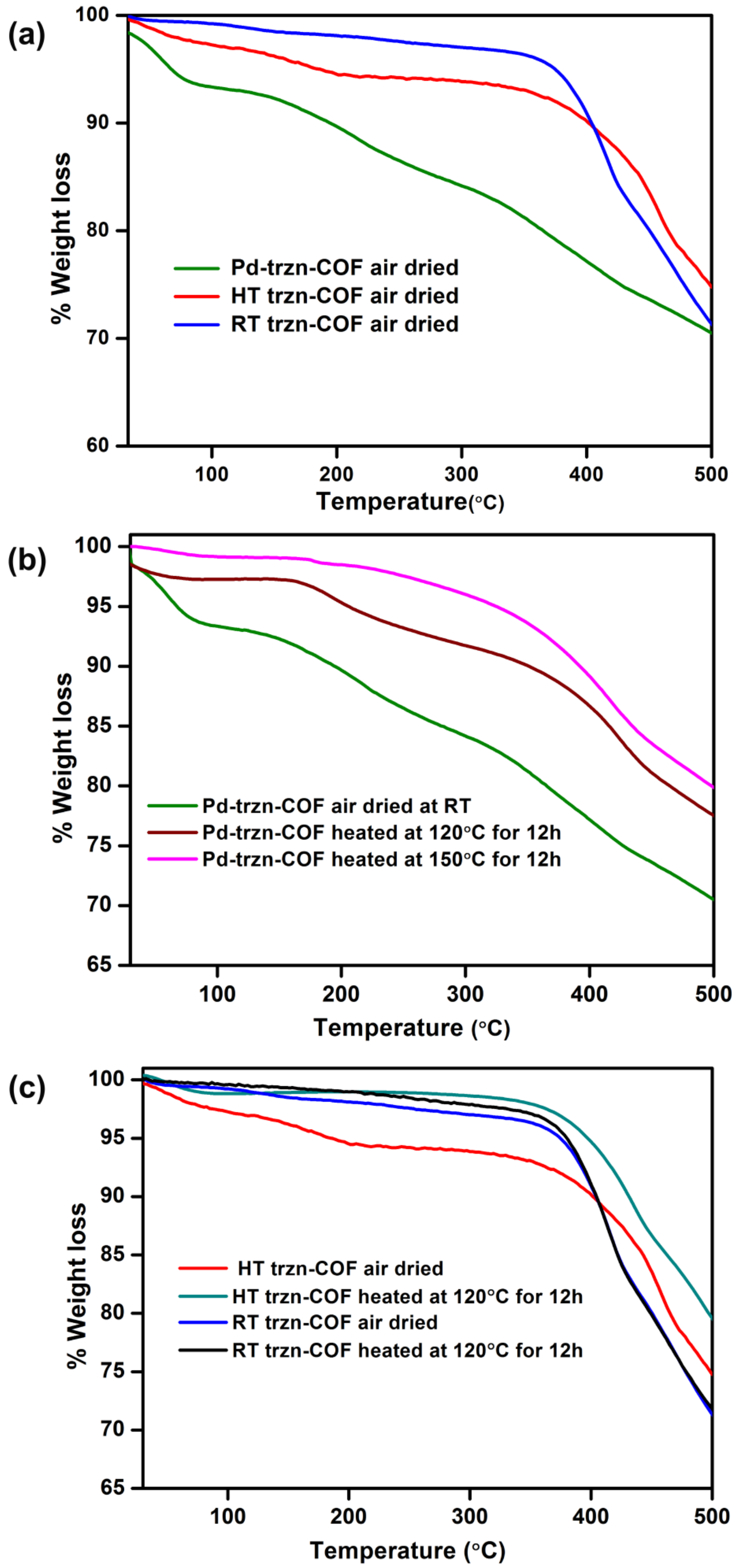


**Figure s2.** Thermogravimetric analysis of trzn-COF and Pd-trzn-COFs. (a) Comparison of the TGAs of trzn-COF and Pd-trzn-COF shows the presence of significant weight loss in the Pd loaded phase and this could be due to the solvent and moisture content that could be strongly adsorbed on the surface of the Pd nano particles. (b) TGA of the high temperature dried Pd-trzn-COF showing the near complete removal of these solvents during this thermal treatment and (c) A comparison of the air dried and high temperature dried samples (under vacuum) showing the facile removal of solvents by heat treatment. The porosity measurements on these heat treated samples (Figure s16) confirmed that the samples are intact upon removal of these solvents at high temperature under vacuum (up to 200$^{o}$C).

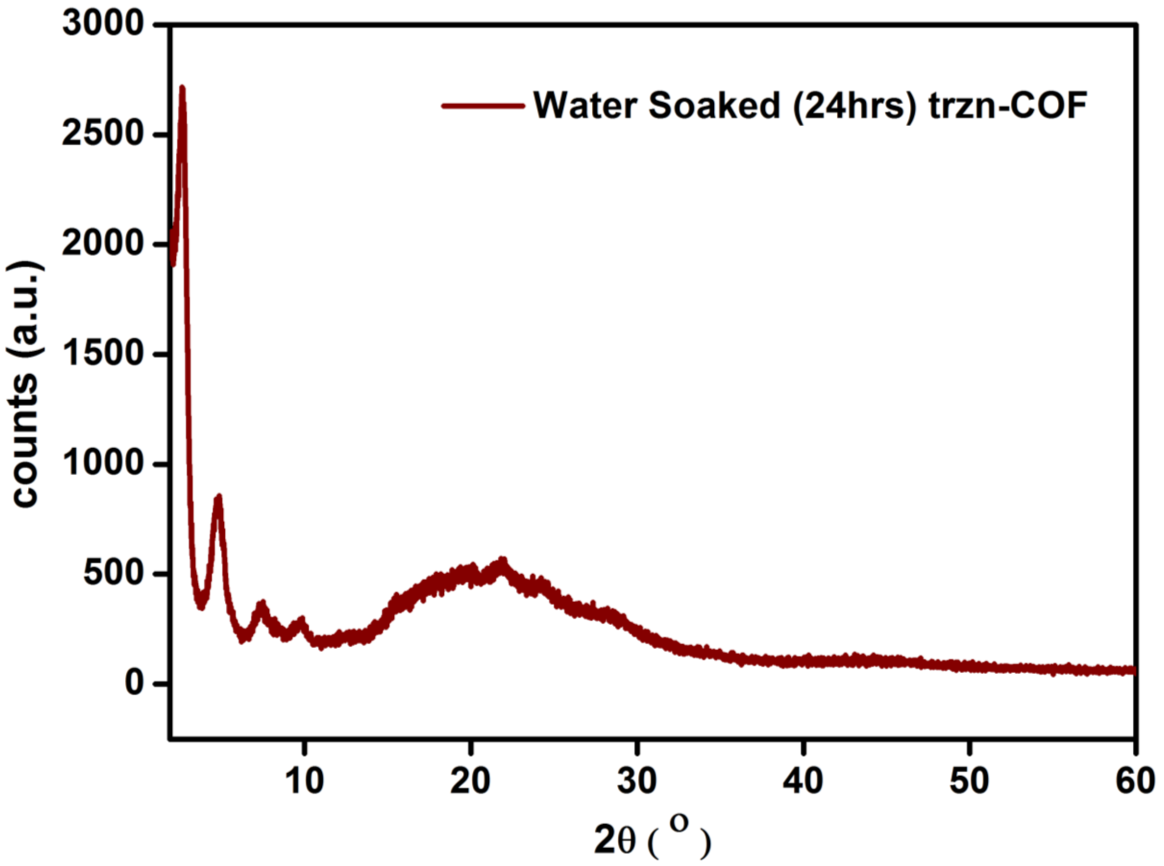


**Figure s3.** Powder pattern of the trzn-COF that was soaked in water for 24hrs at room temperature.

*Crystal coordinates for trzn-COF*

Crystal system: Hexagonal

Space group: P6/mcc

A = 36.3667(2); c = 6.800321(5)

| Atom | Element | x | y | z |
|---|---|---|---|---|
| N1 | N | 0.34466 | 0.63584 | 0.5 |
| C2 | C | 0.30385 | 0.62633 | 0.5 |
| O3 | O | 0.27628 | 0.58348 | 0.5 |
| C4 | C | 0.23327 | 0.56656 | 0.5 |
| C5 | C | 0.20827 | 0.5218 | 0.5 |
| C6 | C | 0.16479 | 0.5026 | 0.5 |
| C7 | C | 0.1457 | 0.52839 | 0.5 |
| C8 | C | 0.1717 | 0.57313 | 0.5 |
| C9 | C | 0.2145 | 0.59211 | 0.5 |
| C10 | C | 0.10366 | 0.51855 | 0.5 |
| N11 | N | 0.06595 | 0.48193 | 0.5 |
| C12 | C | 0.03384 | 0.48968 | 0.5 |
| C13 | C | 0.04126 | 0.53476 | 0.5 |
| C14 | C | -0.01031 | 0.45669 | 0.5 |
| H15 | H | 0.22394 | 0.50281 | 0.5 |
| H16 | H | 0.14498 | 0.46801 | 0.5 |
| H17 | H | 0.15684 | 0.59285 | 0.5 |
| H18 | H | 0.23242 | 0.62658 | 0.5 |
| H19 | H | 0.10094 | 0.54758 | 0.5 |

## *3. Microscopy*

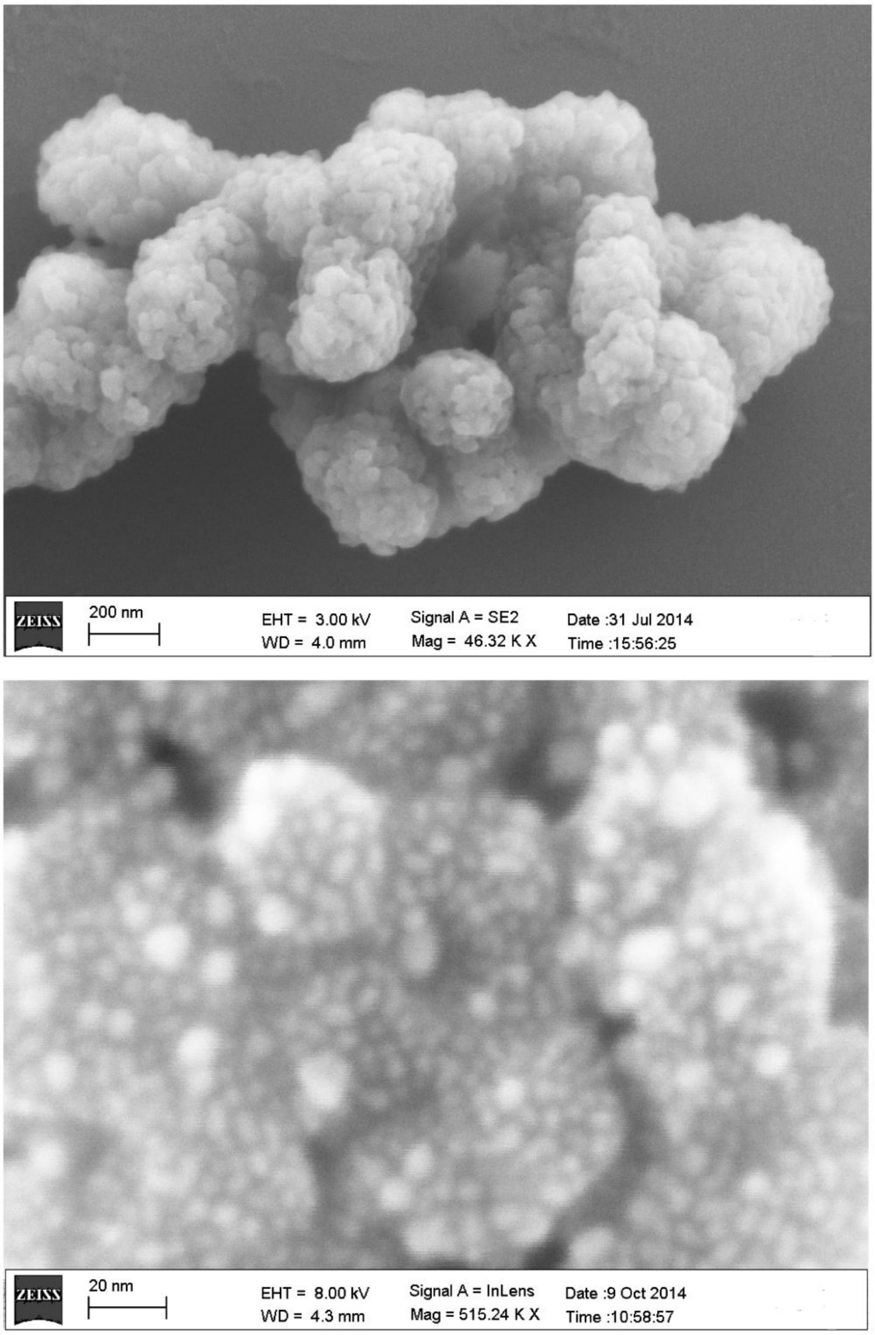


**Figure s4.** SEM image of the as-synthesized trzn-COF, the same sample shown at two different resolutions. From the figure on top, the sample appears to have a homogeneous sphere-like particles. The same when viewed at 20 nm resolution appears to have the flaky appearance with patterned surfaces.

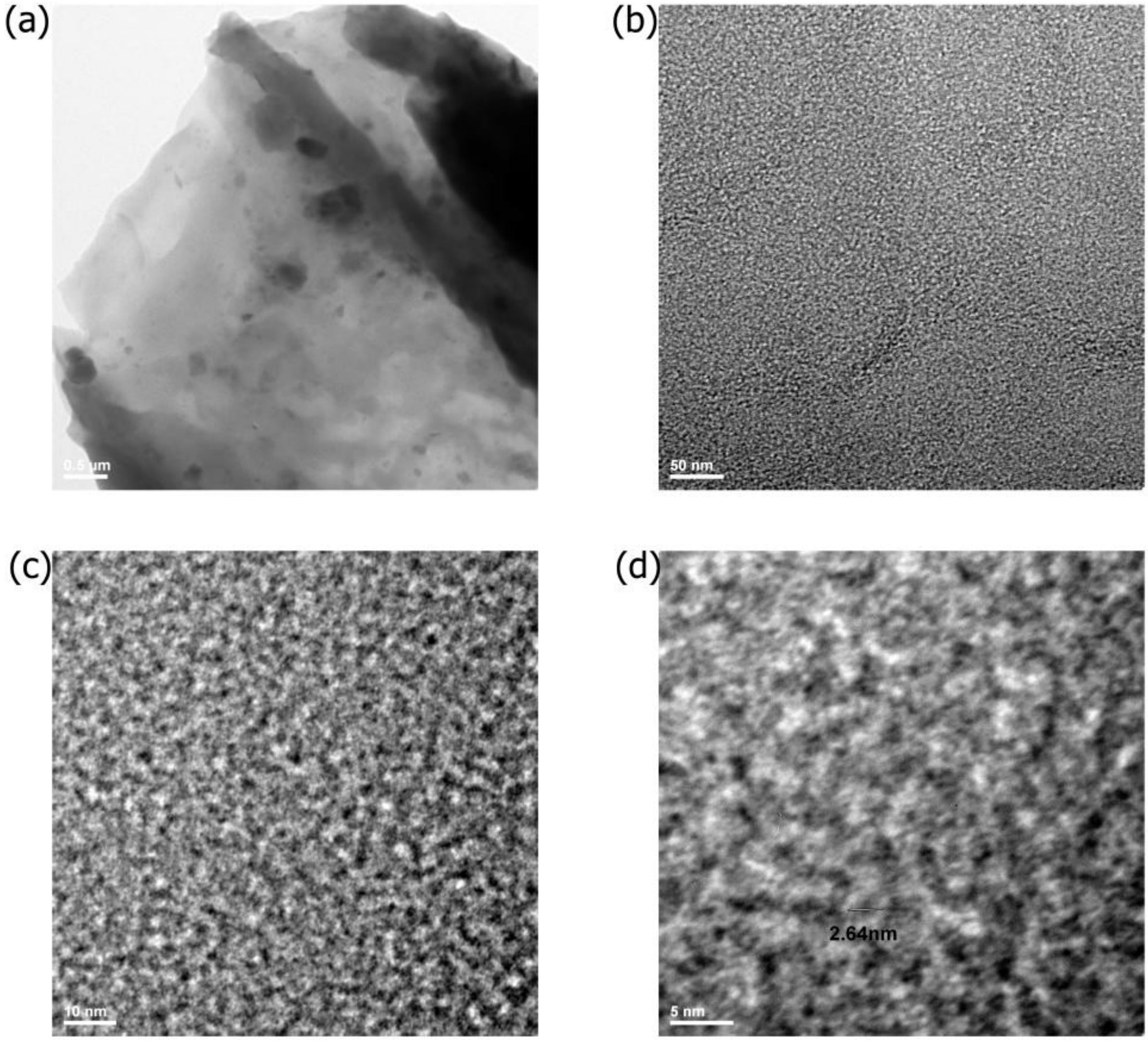


**Figure s5.** TEM images of the as-synthesized trzn-COF (a) showing the transparent layers formed by the COF. (b) the uniform porous texture. (c) and (d) show the HR-TEM of the trzn-COF captured by zooming in on the region shown in figureS5b, at different resolutions, 10 and 5 nm. These clearly show the presence of small pores.

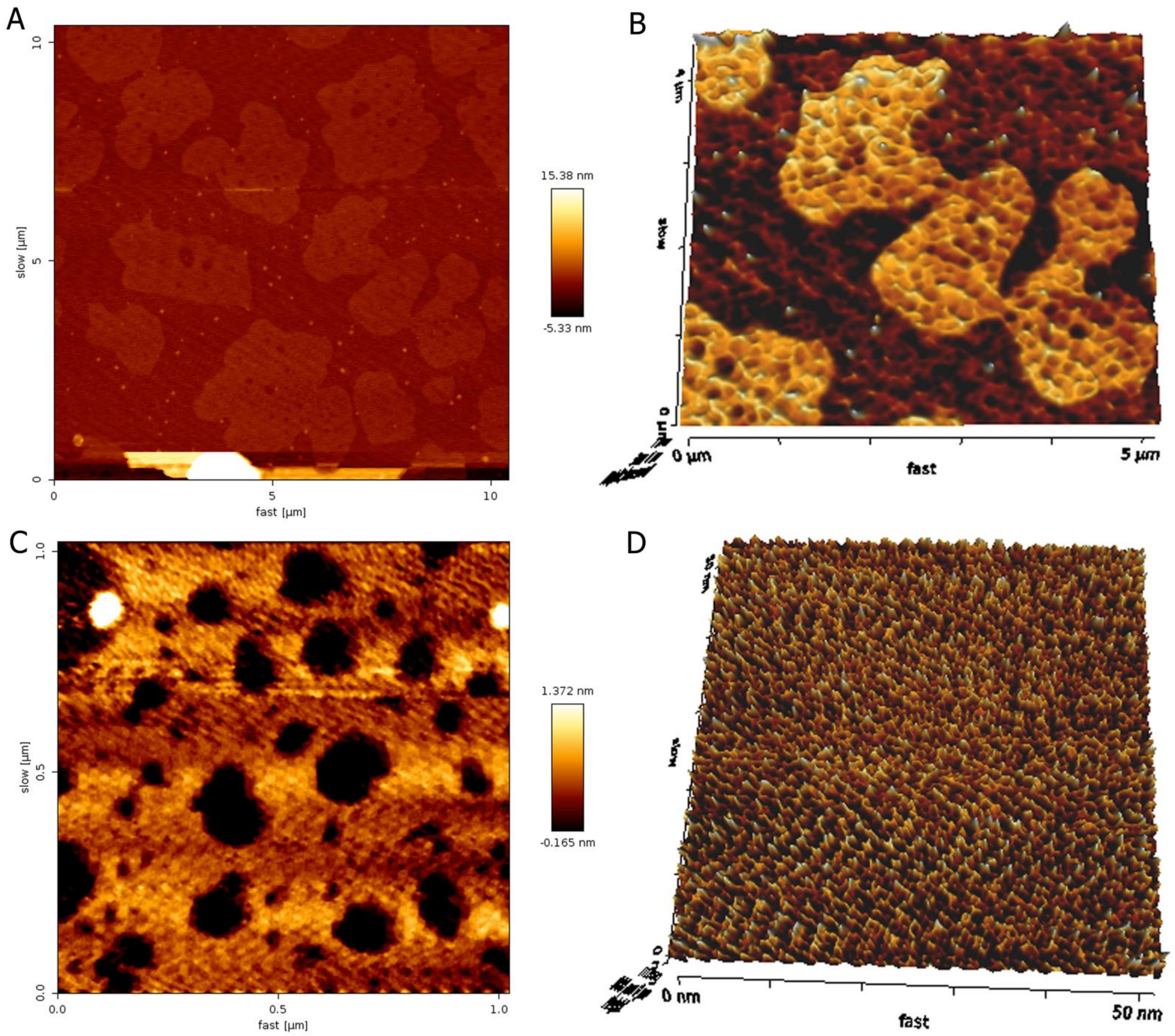


**Figure s6.** AFM images of the trzn-COF with increasing resolution. A) 10 µm resolution showing the islands of COF on the substrate. B) A zoom-in on one of the regions. C) A 1 µm image of the same region displaying the presence of macropores and micrometer or micron sized pores and patterned structures on the COF. D) At nanometre resolution the fine carpet-like patterned surface of the COF can be observed..

## *4. EDAX analysis*

### (a) As made trzn-COF

**Project 1**

Spectrum processing :
Peak possibly omitted : 2.627 keV
Processing option : All elements analyzed (Normalised)
Number of iterations = 5
Standard :
C CaCO3 1-Jun-1999 12:00 AM
N Not defined 1-Jun-1999 12:00 AM
O SiO2 1-Jun-1999 12:00 AM

| Element | Weight% | Atomic% |
|---|---|---|
| C K | 65.09 | 69.99 |
| N K | 15.90 | 14.66 |
| O K | 19.01 | 15.35 |
| Totals | **100.00** | |

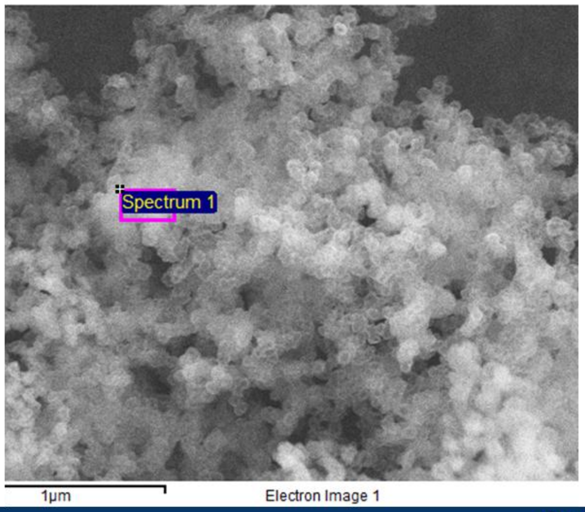


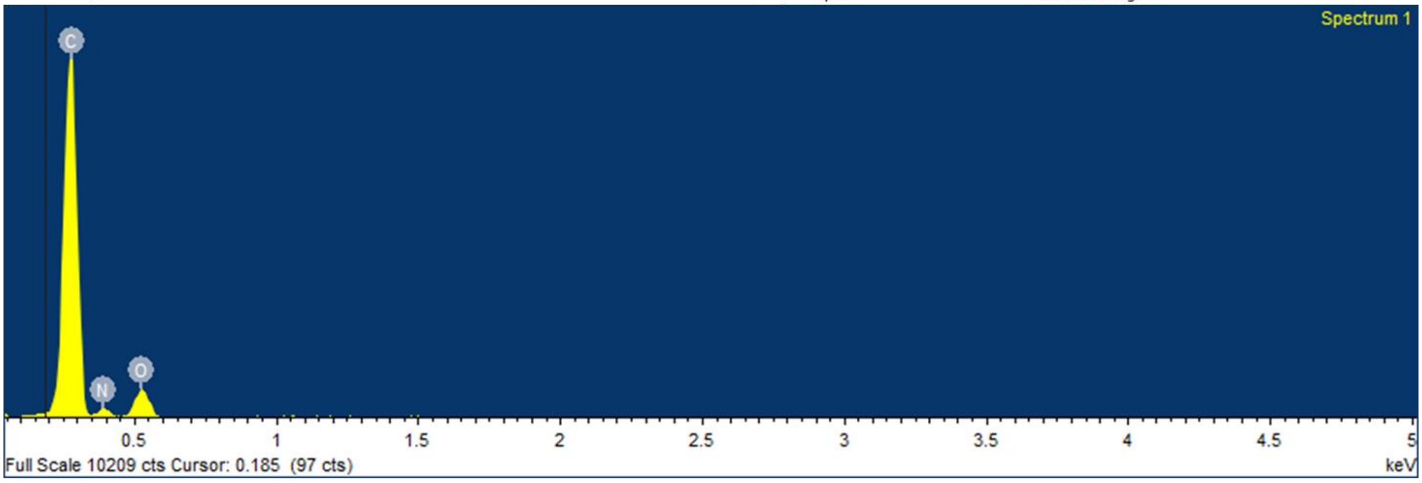

## (b) Pd- trzn-COF

**Project 1** 8/7/2014 5:14:34 PM

Spectrum processing :
Peak possibly omitted : 1.745 keV
Processing option : All elements analyzed (Normalised)
Number of iterations = 4
Standard :
C CaCO3 1-Jun-1999 12:00 AM
N Not defined 1-Jun-1999 12:00 AM
O SiO2 1-Jun-1999 12:00 AM
Pd Pd 1-Jun-1999 12:00 AM

| Element | Weight% | Atomic% |
|---|---|---|
| C K | 42.08 | 54.23 |
| N K | 35.21 | 38.91 |
| O K | 4.33 | 4.19 |
| Pd L | 18.37 | 2.67 |
| Totals | **100.00** | |

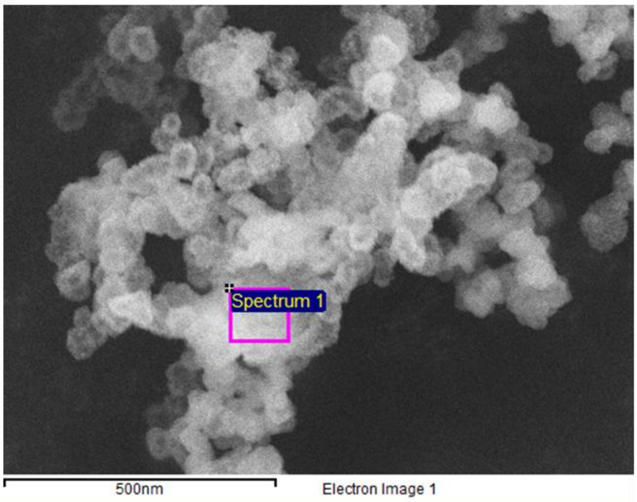


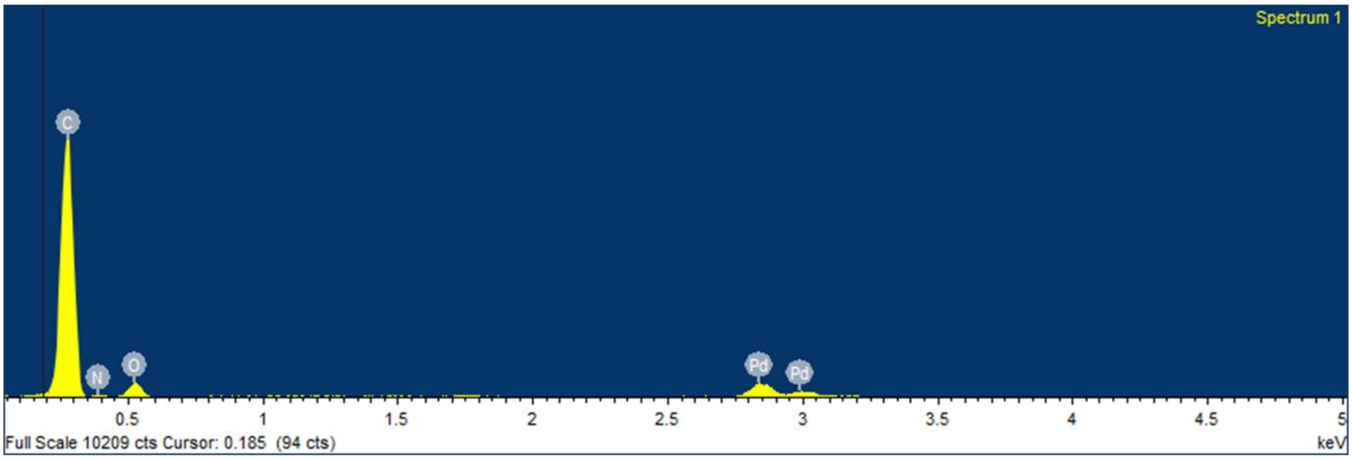


**Figure s7.** EDAX analysis of (a) the trzn-COF and (b) Pd- trzn-COF. Several preparations were screened and the Pd wt% varied between 18-20%.

## *5. X-ray photoelectron spectroscopy*

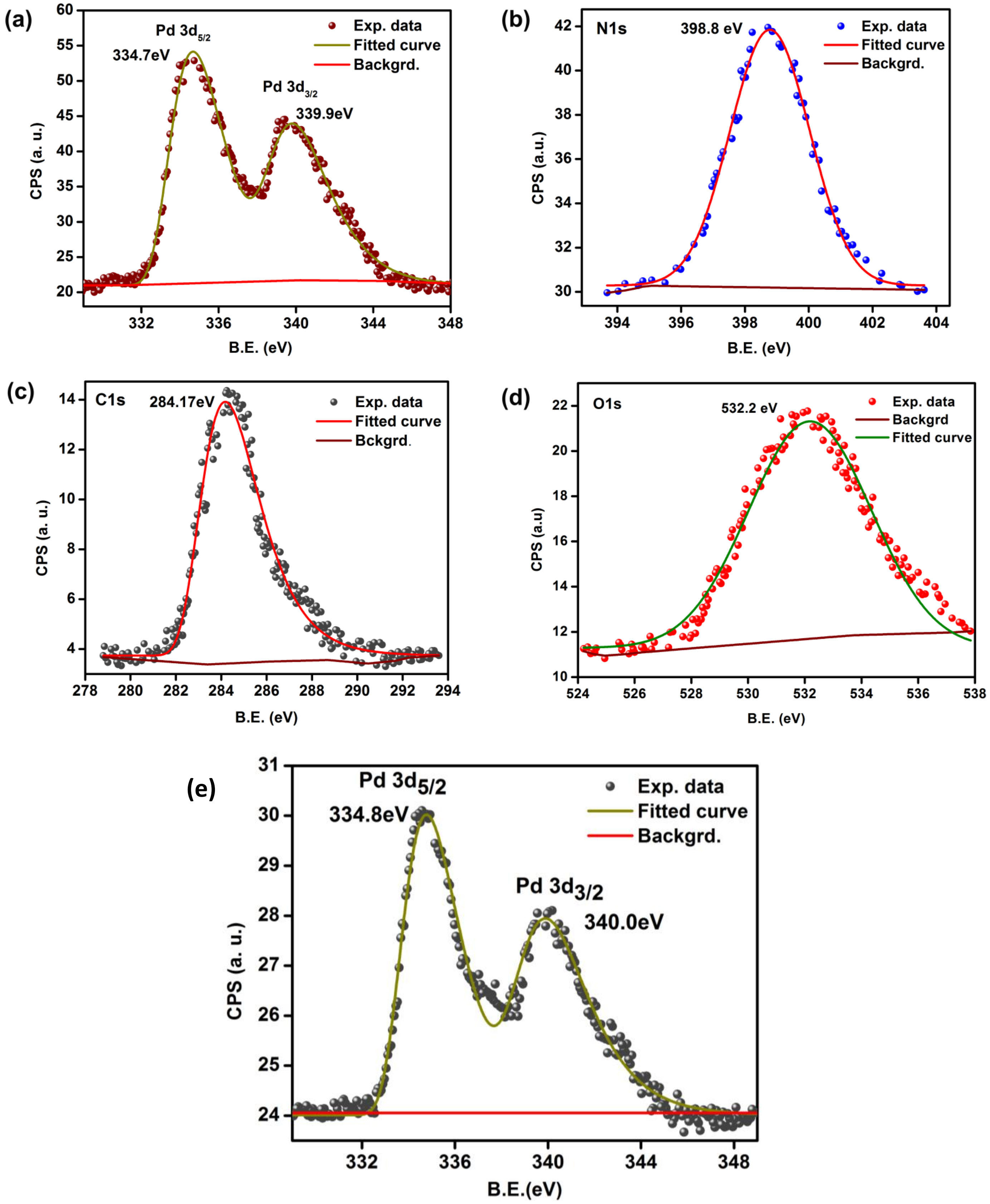


**Figure s8.** XPS spectra of Pd-trzn-COF, (a-d) showing the presence of Pd(0) and the peaks expected for the other elements. The BE observed for the Pd(0) is in good agreement with those found in the literature (Refs: i. Q. L. He et al., *Nat. Scientific Reports*, 3 (**2013**) 2497. ii. T. Arunagiri et al., *Materials Chemistry and Physics* 92 (**2005**) 152. iii. G. B. Hoflund et al., Appl. Surf. Sci., 205 (2003) 102.). (e) Pd(0) peak observed for the spent catalyst.

## *6. Adsorption data*

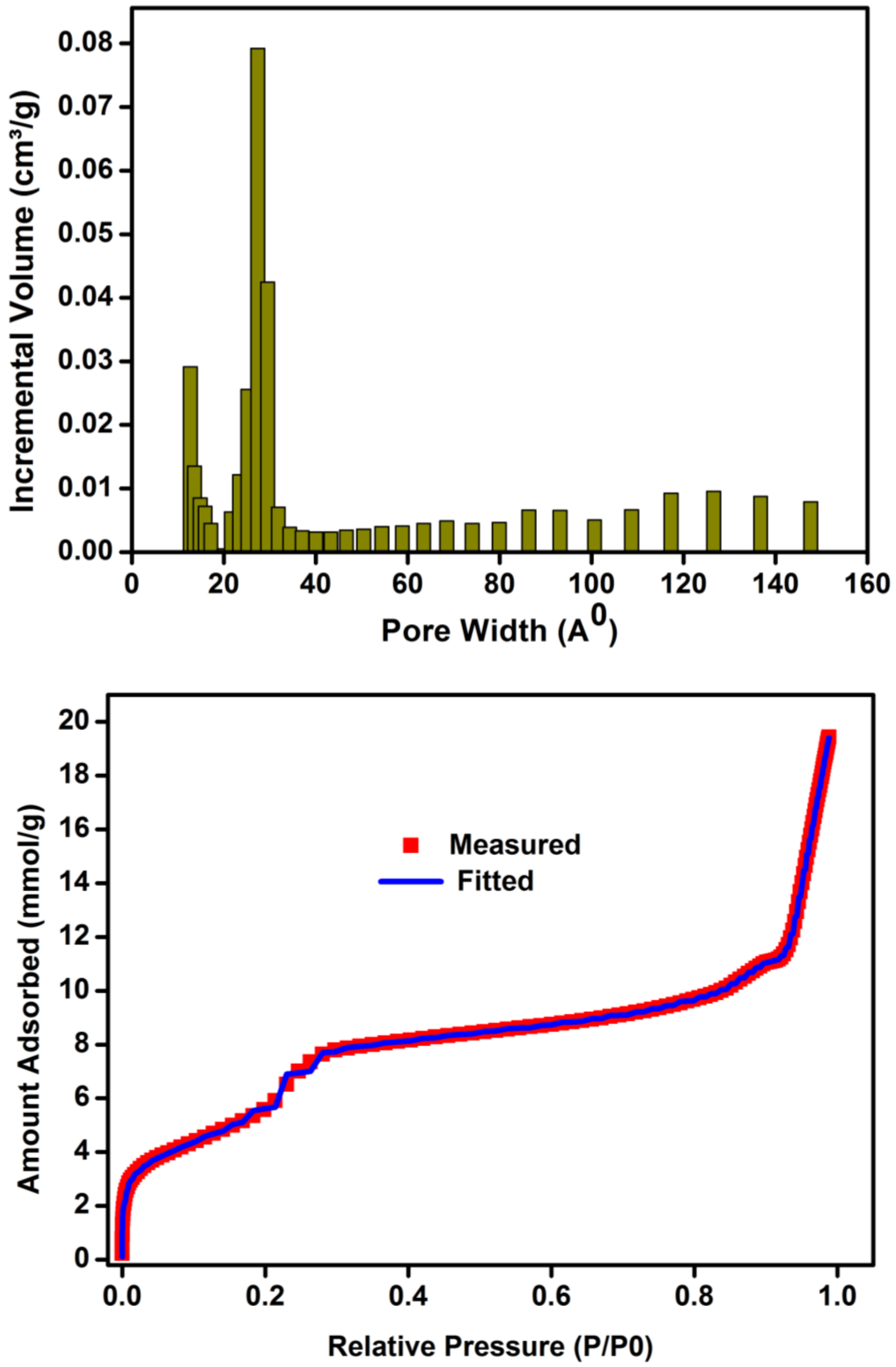


**Figure s9.** Pore size distribution in trzn-COF obtained using a non-localized DFT (NLDFT on carbon) fit carried out on a 77K $N_2$ isotherm. The fit to the low pressure region seem to indicate presence of micropores.

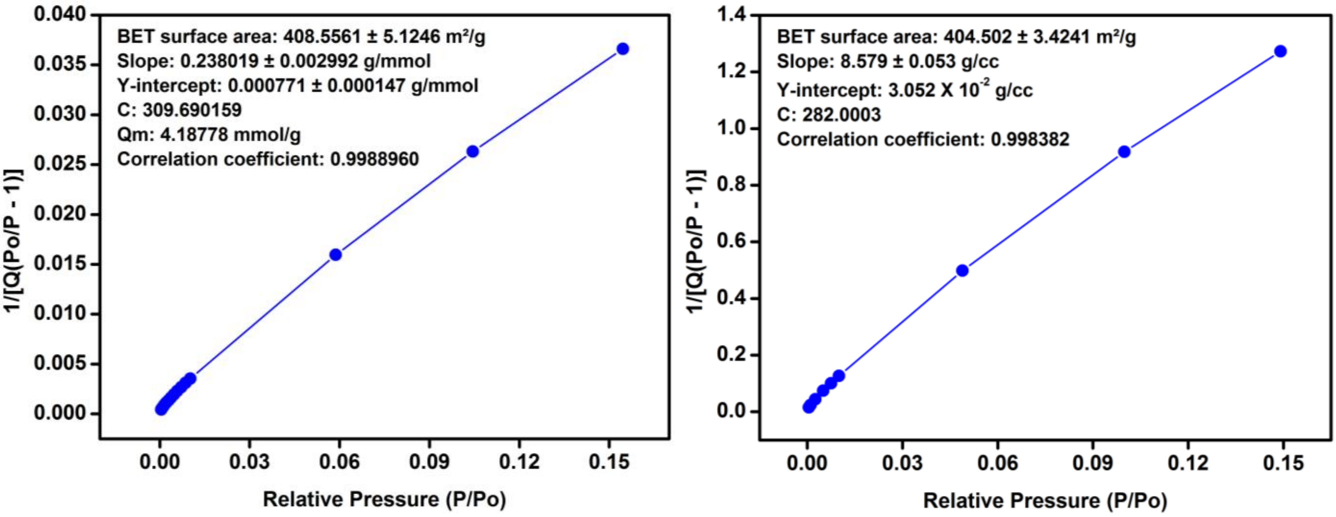


**Figure s10.** Left: A BET fit for as-synthesized trzn-COF, carried out using the 77K $N_2$ isotherm showing a surface area of 408 $m^2$/g, exclusively for the micropore region ($P/P_0$= 0.0001-0.15); the mesoporous region could not be fit satisfactorily with a BET model. Right: The BET fit for the Pd-trzn-COF carried out using the 77K $N_2$ isotherm and in the micropore region showing a surface area of 404$m^2$/g; the mesopores could not be fit satisfactorily using BET. BJH model fitted to the mesoporous region presented a pore size of 23Å, which is in reasonable agreement with the structure. The Dubinin-Radushkevich (DR) model gave a pore volume of 0.21cc/g and a surface area of 563$m^2$/g.

***Discussion on bimodal pore distribution:***

Based on our BET and DFT model based analyses of the 77K $N_2$ isotherm, we do see the presence of these micropores. We realized two possibilities that could create micropores in this material. Considering the very less dense powder character of the material and based on the microscopy images we anticipated some inter-particle spaces that are in the microporous regime. For this reason we prepared this sample differently using methods like mechanical grinding and/or sonication and carried out the $N_2$ adsorptions. All such studies gave a same isotherm profile with an isotherm showing microporous behavior at low $P/P_0$. Another reason could be the high temperature (120$^o$C) synthesis resulting in stacking faults creating micropores, again this is unlikely as we see that the room temperature synthesis where such stacking faults are expected to be minimal also shows this typical micro-mesoporous isotherm profile. This indicates that these pores are inherent to the material. However, now we have used other models (BJH and DR) to describe the pore size distribution, which seem to yield a single mesopores, but they are not capable of fitting the micropores.

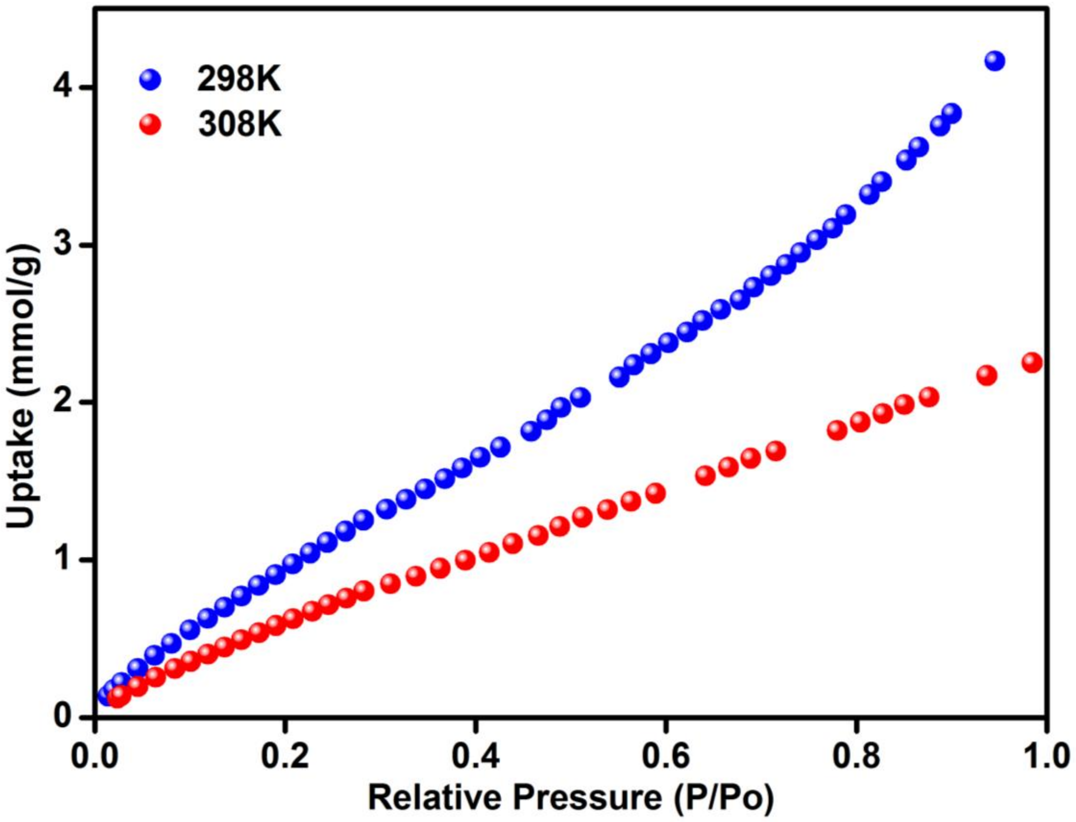


**Figure s11.** Sorption isotherm of water obtained at 298K and 308K and were used for the estimation of HOA for water on trzn-COF.

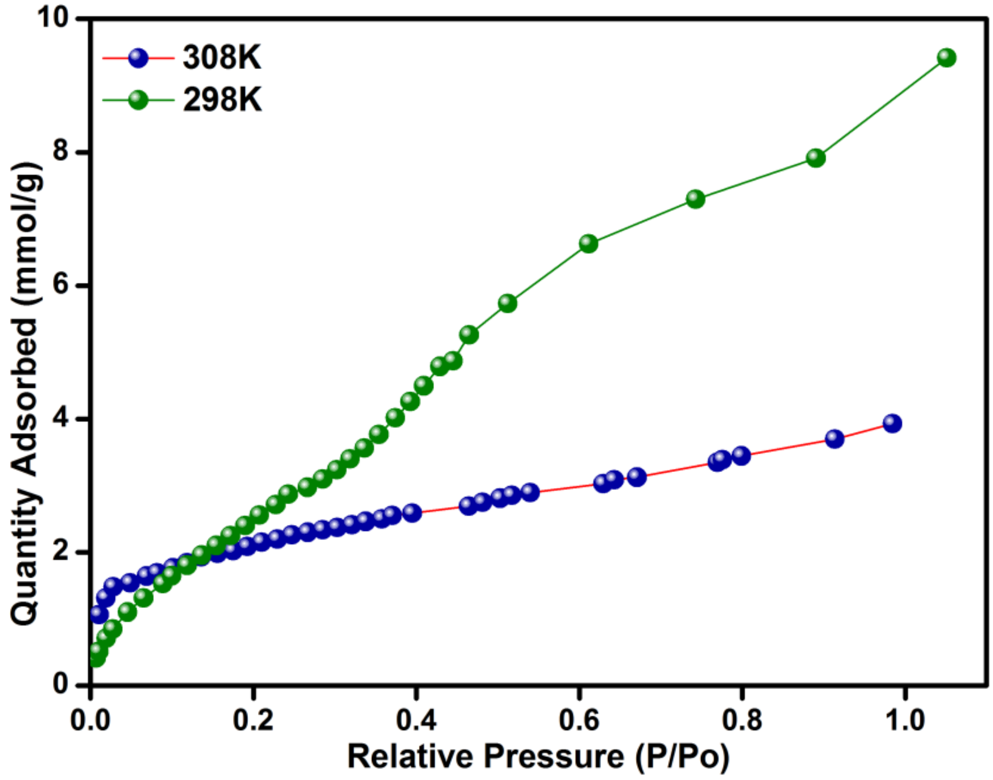


**Figure s12.** Sorption isotherm of methanol obtained at 298K and 308K and were used for the estimation of HOA for methanol on trzn-COF.

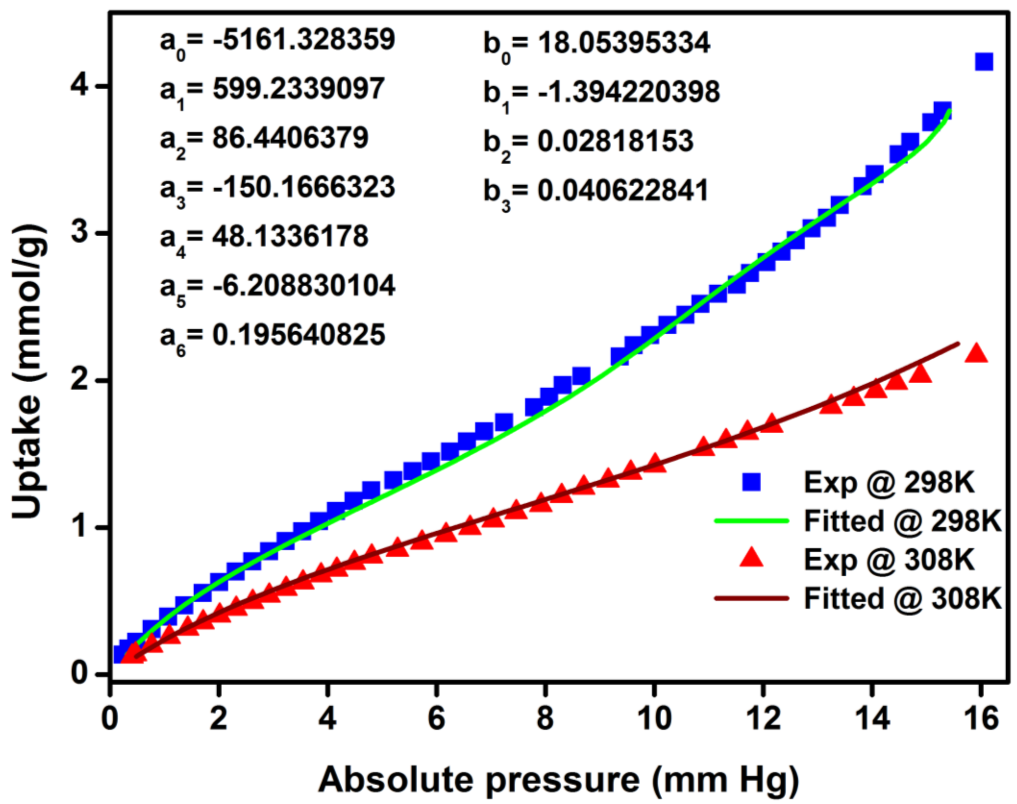


**Figure s13.** Virial fit parameters employed for fitting the Water isotherm data.

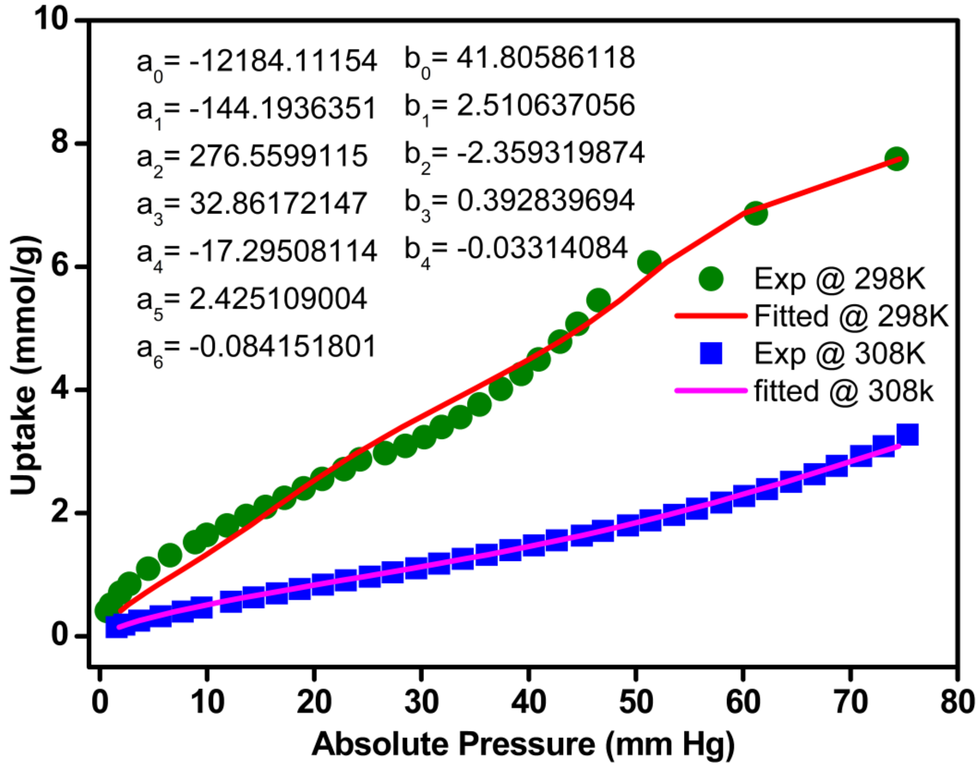


**Figure s14.** Virial fit parameters employed for fitting the MeOH isotherm data.

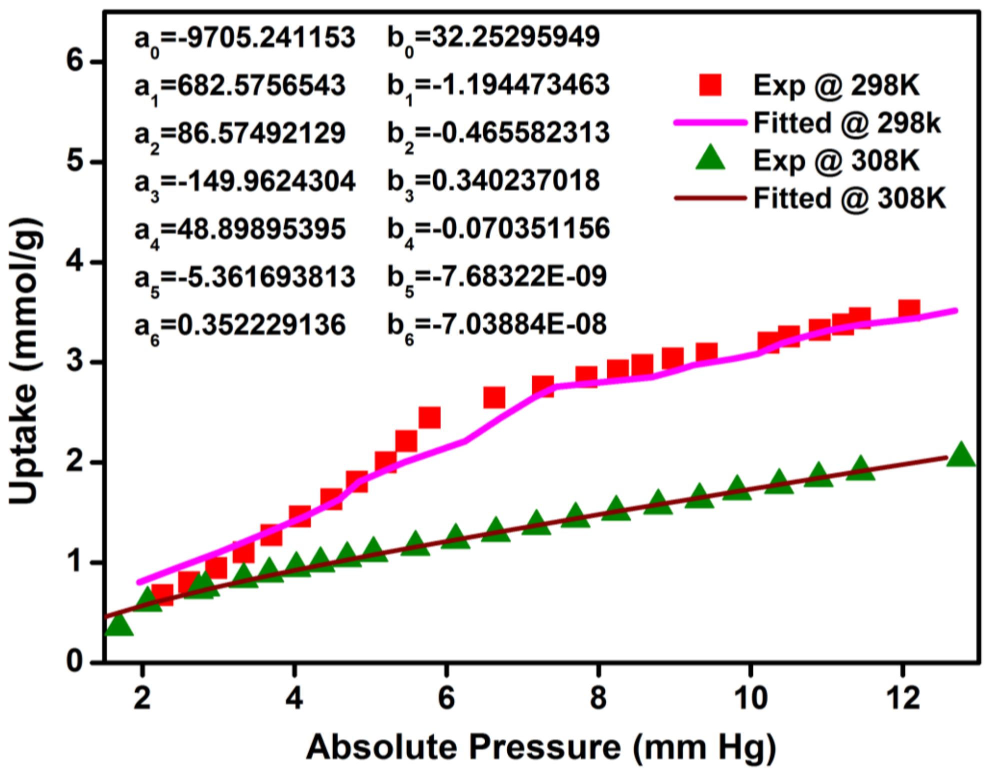


**Figure s15.** Virial fit parameters employed for fitting the Toluene isotherm data.

***Stability of the Pd-trzn-COF to heat treatments:***

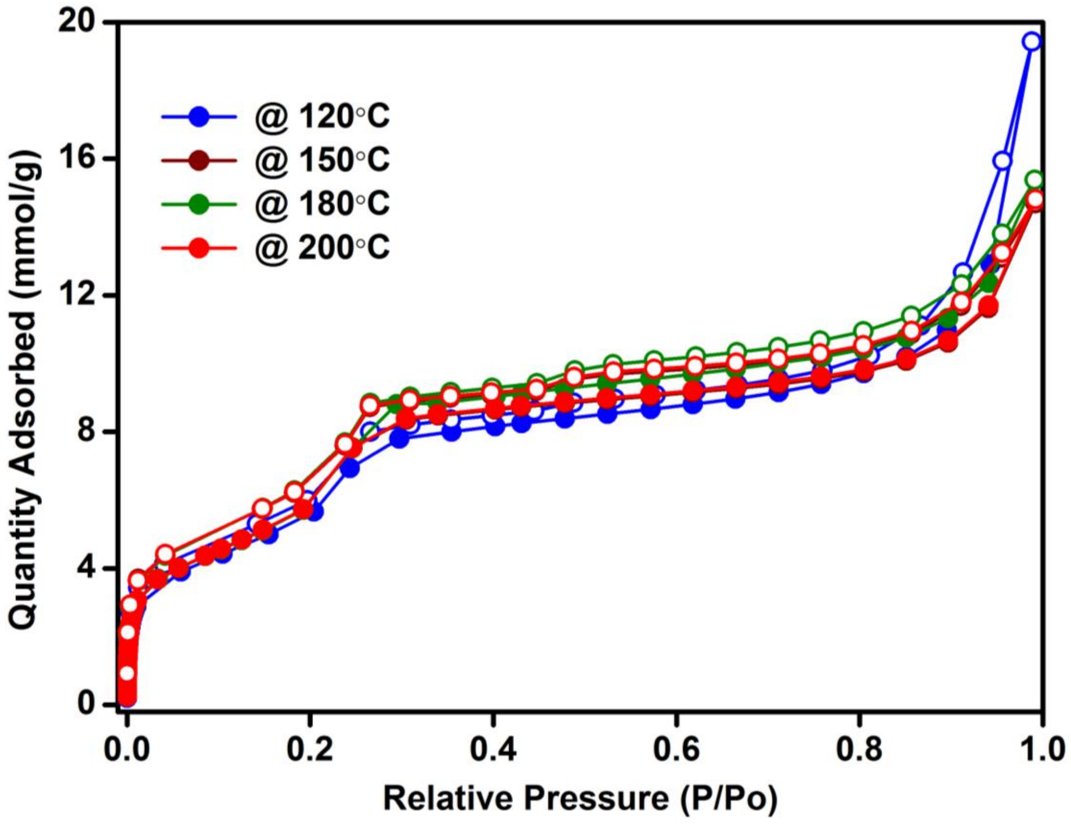

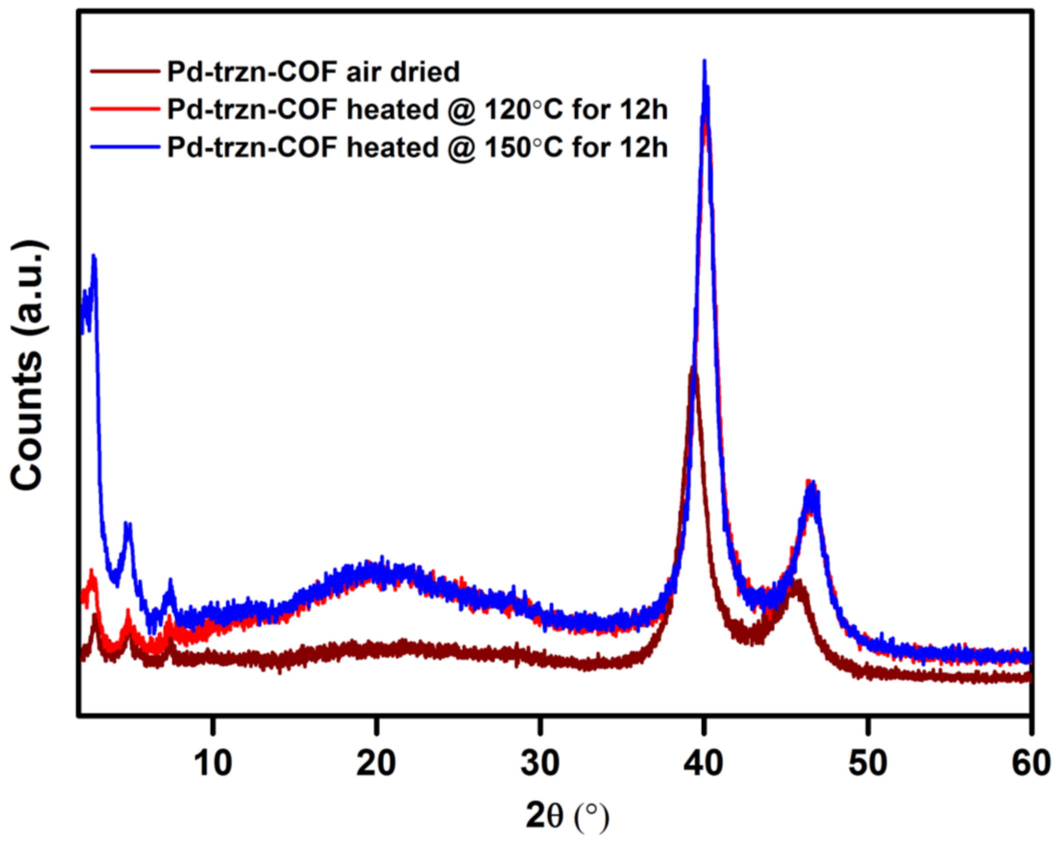


**Figure s16.** Shows the 77K $N_2$ adsorption isotherms of Pd-trzn-COF heat treated at different temperatures under vacuum atmosphere. The PXRDs showing their crystallinity. Note, there is no change in the profiles or uptakes of the isotherms.

***Supporting discussion on lack of pore size reduction with Pd loading:***

It is quite unusual trzn-COF did not lose any $N_2$ uptake on $Pd^0$ loading unlike other $Pd^{2+}$ loaded COFs. This could suggest the presence of only $Pd^0$ in our COF as it occupies much lesser pore space than $Pd(OAc)_2$ . Adding the $Pd^0$ to the weight of the adsorbing sample should be expected to bringing down the total $N_2$ uptake? One possible explanation would be that the surface grafted $Pd^0$ also acts as a $N_2$ adsorption site, which would add to the overall $N_2$ uptake and purely by coincidence this matches up with the porosity of the pristine COF. Also, significant amount of the Pd nanoparticles are much larger than the pore size (2.7nm) and are located on the surface of the COF as observed from the FE-SEM and HR-TEM. These particles most likely do not seem to hinder the access to the pores, in which case, again no significant drop in porosity would be expected on Pd loading. Importantly, when a model independent BJH method is used, we obtain mesopores of size 23Å for the as-made form, trzn-COF and this drops down to 19Å for the Pd loaded phase, Pd-trzn-COF. This drop could certainly be attributed to the loading of some small (< 3nm) Pd nanoparticles into the pore.

## *7.Catalysis: Heck coupling catalysis*

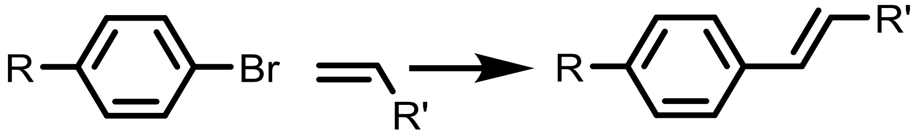


**Table s1.** Heck reactions on substrates with electron withdrawing or electron donating substituents

| Entry | R | R’ | % yield | TON | TOF($h^{-1}$) |
|---|---|---|---|---|---|
| 1 | H | $CO_2$n-Bu | 82 | 2473 | 2473 |
| 2 | $CH_3$ | $CO_2$n-Bu | 85 | 2394 | 2394 |
| 3 | $OCH_3$ | $CO_2$n-Bu | 80 | 2262 | 2262 |
| 4 | CN | $CO_2$n-Bu | 92 | 2420 | 2420 |
| 5 | $NO_2$ | $CO_2$n-Bu | 93 | 2446 | 2446 |
| 6 | H | Ph | 85 | 2500 | 2500 |
| 7 | $OCH_3$ | Ph | 88 | 2315 | 2315 |
| 8 | CN | Ph | 88 | 2288 | 2288 |
| 9 | $NO_2$ | Ph | 91 | 2394 | 2394 |

Amount of catalyst used in each reaction = 1mg for 1.0mmol of reactant, reaction time1hr at 120$^{o}$C, Aryl halide (1.0 mmol), butyl acrylate or styrene (1.1 mmol), sodium acetate (1.2 mmol), and Pd-trznCOF (~0.01 mol%) were added to 3ml of N-Methyl-2-pyrrolidone. % of yield = (Actual weight of the yield/ predicted weight of the product)∗100 ; TON (Turnover number) calculation = moles converted / moles of active sites; TOF ( Turnover frequency) = TON/Time hours. Note the wide range of substrates with aromatic and aliphatic substituents.

## *8. C-C Homo Coupling*

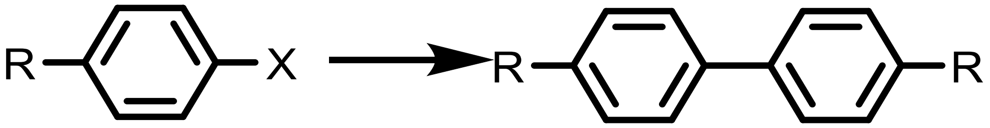


**Table s2.** C-C coupling reactions on substrates with electron withdrawing or electron donating substituents

| Entry | X | R | %yield | TON | TOF($h^{-1}$) |
|---|---|---|---|---|---|
| 1 | I | H | 93 | 611 | 102 |
| 2 | Br | H | 90 | 591 | 99 |
| 3 | I | $OCH_3$ | 86 | 565 | 94 |
| 4 | Br | $OCH_3$ | 80 | 525 | 88 |

| | | | | | |
|---|---|---|---|---|---|
| **5** | **I** | **$COCH_3$** | **93** | **611** | **101** |
| **6** | **I** | **$COOCH_3$** | **95** | **624** | **104** |
| **7** | **Br** | **$COCH_3$** | **87** | **571** | **95** |
| **8** | **Br** | **$COOCH_3$** | **92** | **604** | **101** |

Amount of catalyst used in each reaction = 4mg for 1.0mmol of reactant, Aryl halide (0.5mmol), Potassium Carbonate (0.6mmol),and Pd-trzn-COF (2mg,) were added to 3ml of DMF. The reaction mixture was stirred at 120$^{o}$C for 6hrs in open air.

### *9. C-C Heterocoupling (Suzuki couplings)*

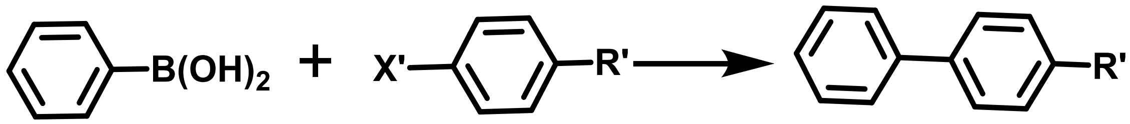


**Table s3.** C-C coupling reactions on substrates with electron withdrawing or electron donating substituents

| **Entry** | **X** | **R** | **%yield** | **TON** | **TOF($h^{-1}$)** |
|---|---|---|---|---|---|
| **1** | **I** | **H** | **99** | **2604** | **651** |
| **2** | **Br** | **H** | **98** | **2578** | **645** |
| **3** | **I** | **COMe** | **99** | **2604** | **651** |
| **4** | **Br** | **COOMe** | **99** | **2604** | **651** |
| **5** | **I** | **NO2** | **99** | **2604** | **651** |
| **6** | **Br** | **OMe** | **99** | **2604** | **651** |

Aryl halide (1.0 mmol), phenylboronic acid (1.1 mmol), NaOH (1.2 mmol), Tetra-n-butylammonium bromide (TBAB) (1.2 equiv), 0.01mol% of Pd- trzn-COF were added to 3mL of water. Contents were stirred at 65$^{o}$C for 4hrs in open air.

***Additional test to confirm lack of Pd leaching:***

In a separate experiment, the Pd-trzn-COF was stirred in DMF solution at 120ºC for 1hr and when the supernatant was isolated and evaporated to dryness, and analyzed using EDAX it showed nil Pd redundant. Importantly, this extract was suspended back into DMF and it was employed for Rxn. 5 in the table s1 and no product was obtained.

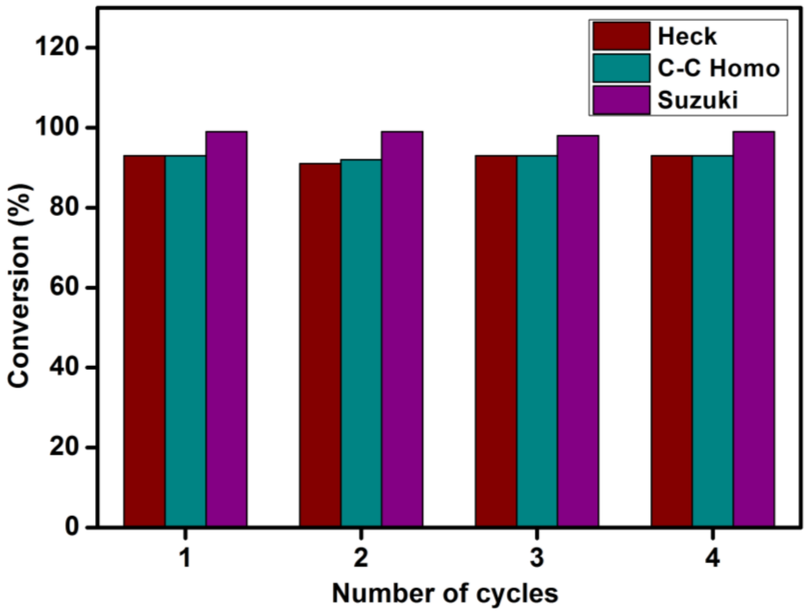


**Figure s17.** A bar chart showing the yield of products obtained via either Heck, non-boronic acid based C-C homo coupling and boronic acid based Suzuki coupling using some representative reactions. In these recycle tests, a reaction involving p-nitrobromobenzene (101 mg, 0.5 mmol), n-butylacrylate (74 mg, 0.575 mol), sodium acetate (51mg, 0.625 mmol), and Pd-trzn-COF (0.5 mg) in 3mL of NMP was used as a representative.

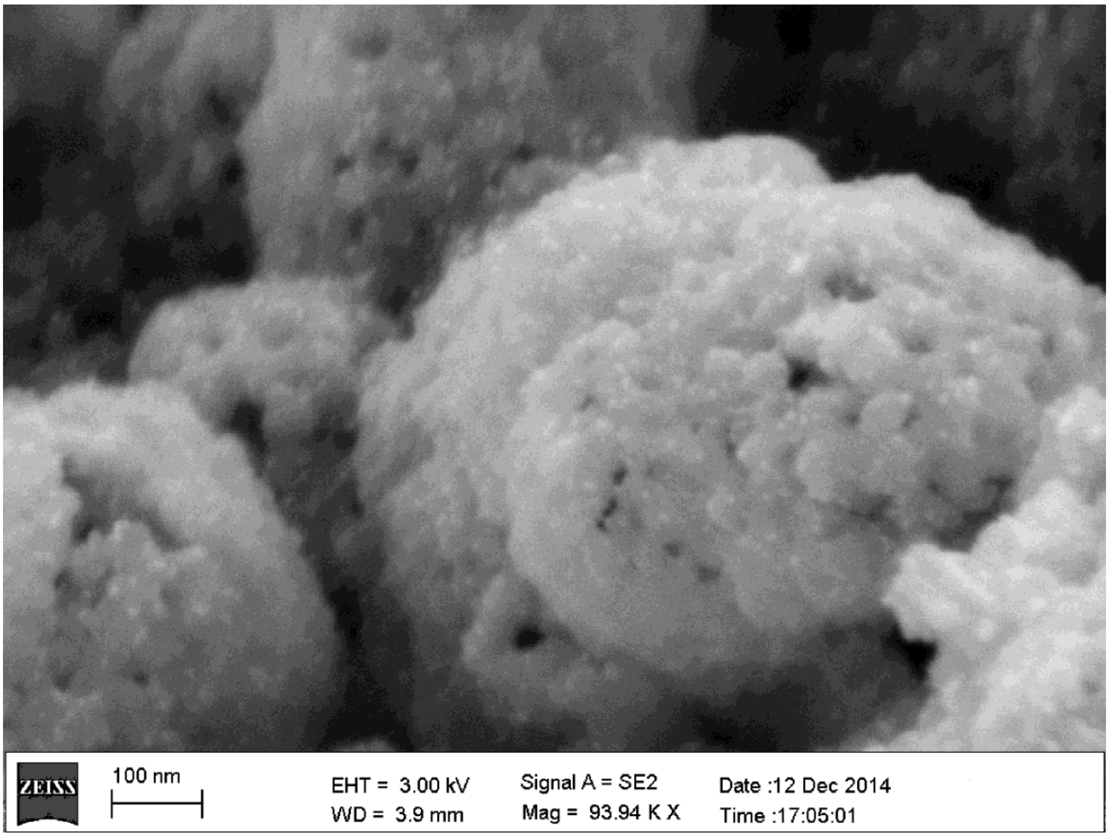


**Figure s18.** SEM image of the Pd-trzn-COF post CO oxidation reaction. Note the fine dispersion of the Pd nanoparticles and there seems to be no sintering.

***Interactions of Pd-trzn-COF with $H_2$ and $O_2$:***

We carried out these measurements to screen for any type of chemisorption at these temperatures. H2 (77 and 303K) and O2 (273 and 303K) adsorptions were carried out on Pd-trzn-COF. No adsorption was observed for $O_2$ and for $H_2$ very little physisorptive uptake (~1 mmol/g) was observed only at 77K and none at RT. Also, the material showed no degradation upon exposure to these gases during the adsorption. Also, the sample exposed to oxygen during the CO oxidation experiments was intact, confirming the stability of the sample to oxygen even at elevated temperatures. Thus indicating an apparent stronger chemical interactions with CO over these gases. Note that the interaction with CO is claimed from the oxidation experiment.

## 8. trzn-COF-PMMA and Pd-trzn-COF-PMMA composites

A major concern with Schiff base COFs is the hydrolyzable nature of the Schiff bonds under both acidic and basic conditions or even when subjected to heating in aqueous solutions. Banerjee and co-workers developed a COF based on keto-enol tautomerism,[37] which has shown exceptional chemical stabilities to date. However, implementing this chemistry in many monomeric units is not easy or would be expensive. In fact, the starting materials for their COF is quite expensive. This brings forth the need for development of other strategies for improving the stability of these Schiff based COFs. Since covalent organic frameworks are built up entirely from organic components they should poses inherent characteristics to blend with other organic materials.

**PMMA-COF composite preparation**: In a typical synthesis of the composite, the trzn-COF (50mg) or Pd-trzn-COF (50gm) was dispersed in THF solution (5mL) containing PMMA (50mg). Membranes were made by spin coating these dilute dispersions at 60°C. A loading of up to 50% could be achieved. The resulting composite can be made into membranes of different shape and size. Contact angle of the membrane was measured to be in the range of 110-120°. *The presence of the hydrophobic casing could favor longer life of the catalyst, and facilitate easy handling and recovery*.

Unfortunately, the PMMA part of the composite is incompatible with DMF. To tackle this issue, we have managed to use the membrane tablets of the composite in methanol and carry out the catalysis for Rxn. 5 in Table s1. This yielded the same product with similar yields indicating no loss in activity of the catalyst membrane. The catalyst-PMMA membranes showed no sign of Pd leaching during catalysis. However, significant swelling of the membrane was observed in methanol.

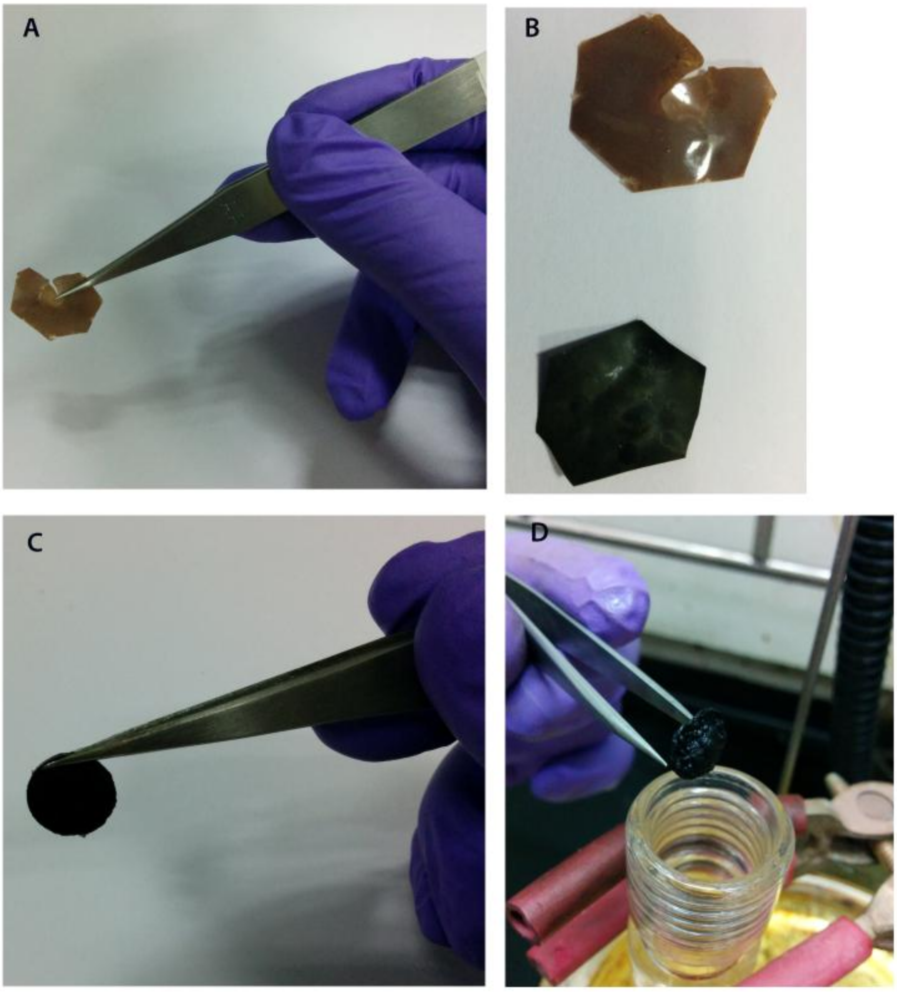


**Figure s19.** (A) A film of the PMMA-trzn-COF composite, thickness=0.2 mm. Note the homogeneity of loading is quite high. (B) Comparison of the film formed using PMMA-trzn-COF and PMMA-Pd-trzn-COF. The Pd-trzn-COF could be loaded at various weight percentage ranging from 20 to 50%. They retained the smooth membrane character even at 50%. (C) shows the membrane-tablet (thickness= ~0.6mm) made by pressing the composite. (D) shows the post catalysis membrane-tablet and also demonstrates the facile handling. A mass balance and EDAX analysis indicated that none of the pellet was lost during the catalysis, confirming lack of any $Pd^0$ leaching.

# Appendix

**Multiple Bromo activation**

## Butyl cinnamate (1a)

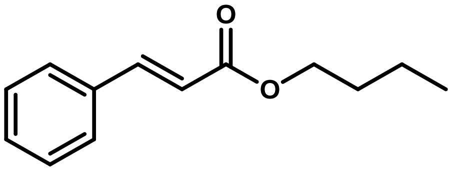

$^{1}H$ NMR (400 MHz, CDCl3, TMS): δ 7.68 (d, J = 16.0 Hz, 1H), 7.54 – 7.48 (m, 2H), 7.37 (dd, J = 4.0 Hz, 3H), 6.43 (d, J = 16.0 Hz, 1H), 4.20 (t, J = 7.1 Hz, 2H), 1.72 – 1.65 (m, 2H), 1.48 – 1.38 (m, 2H), 0.96 (t, J = 7.4 Hz, 3H).

$^{13}C$ NMR (100MHz, CDCl3, TMS): δ 167.50, 144.95, 134.87, 130.61, 129.27, 128.45, 118.68, 64.83, 31.18, 19.61, 14.16.

HRMS: calcd for $C_{13}H_{16}O_2$ $[M+Na]^+$ 227.1047, found 227.1051.

### (E)-1,2-Diphenylethene (1b)

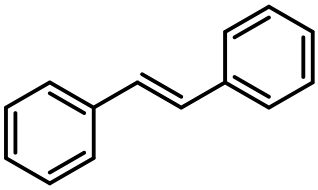

$^{1}H$ NMR (400 MHz, CDCl3, TMS): δ 7.58-7.60 (m, 4H), 7.41-7.45(m, 4H), 7.32-7.35(m, 2H), 7.19 (s, 2H).

$^{13}C$ NMR (100 MHz, CDCl3, TMS): δ 137.43, 128.79, 127.73, 126.64.

HRMS: calcd for $C_{14}H_{12}Na$ $[M+Na]^+$ 181.1017, found 181.0908

### Dibutyl 3,3'-(1,4-phenylene)(2E,2'E)-diacrylate (2a)

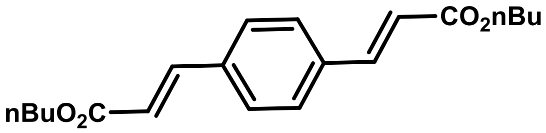


$^{1}H$ NMR (400 MHz, CDCl3, TMS): δ 7.65 (d, J = 16.0 Hz, 2H), 7.53 (s, 4H), 6.47 (d, J = 16.0 Hz, 2H), 4.21 (t, J = 6.7 Hz, 42H), 1.79 – 1.59 (m, 4H), 1.48 – 1.38 (m, 4H), 0.96 (t, J = 7.4 Hz, 6H).

$^{13}C$ NMR (100 MHz, CDCl3, TMS): δ 166.20, 142.73, 135.49, 127.83, 118.69, 63.91, 30.09, 18.53, 13.09.

## 1,4-Di((E)-styryl)benzene (2b)

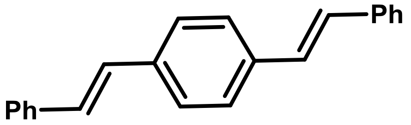


$^{1}$H NMR (400 MHz, CDCl3, TMS): δ 7.55 – 7.48 (m, 8H), 7.37-7.33 (m, 4H), 7.28 – 7.22 (m, 2H), 7.12 (s, 4H)

$^{13}$C NMR (400 MHz, CDCl3, TMS): δ 137.36, 136.74, 128.74, 128.63, 128.30, 127.69, 126.88, 126.55.

HRMS

HRMS: calcd for C14H19N3ONa [M +Na]+ 227.1047, found 227.1051.

## Tributyl 3,3',3''-(benzene-1,3,5-triyl)(2E,2'E,2''E)-triacrylate (3a)

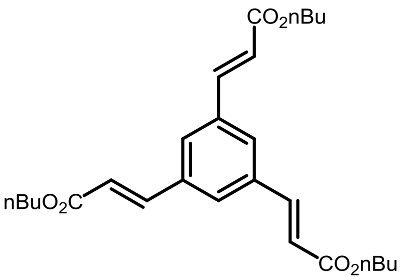


$^{1}$H NMR (400 MHz, CDCl3, TMS): δ 7.54 (m, 6H), 6.40 (d, J = 16.0 Hz, 3H), 4.10 (t, J = 6.7 Hz, 6H), 1.73 – 1.47 (m, 6H), 1.37 – 1.28 (m, 6H), 0.85 (t, J = 7.4Hz, 9H).

$^{13}$C NMR (100 MHz, CDCl3, TMS): δ 166.03, 142.36, 135.46, 128.17, 119.77, 64.19, 30.34, 18.80, 13.34.

HRMS: calcd for $C_{27}H_{36}O_6H[M+H]^+$ 457.2590, found 457.2591

## 1,3,5-Tri((E)-styryl)benzene (3b)

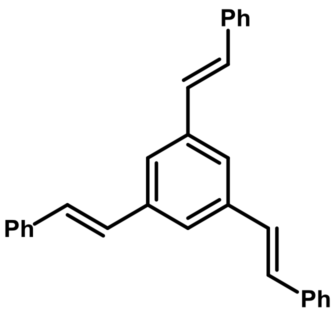


$^{1}$H NMR (400 MHz, CDCl3, TMS): δ 7.57-7.55 (m, 9H), 7.39 (t, *J* = 7.5 Hz, 6H), 7.29 (t, *J* = 7.3 Hz, 3H), 7.23 (A of ABq, J = 16 Hz, 3H),7.18 (B of ABq, J = 16 Hz, 3H)

$^{13}$C NMR (100 MHz, CDCl3, TMS): δ 138.13, 137.34, 129.37, 128.86, 128.47, 127.89, 126.72, 124.09.

## Tetrabutyl 3,3',3'',3'''-(pyrene-1,3,6,8-tetrayl)(2E,2'E,2''E,2'''E)-tetraacrylate (4a)

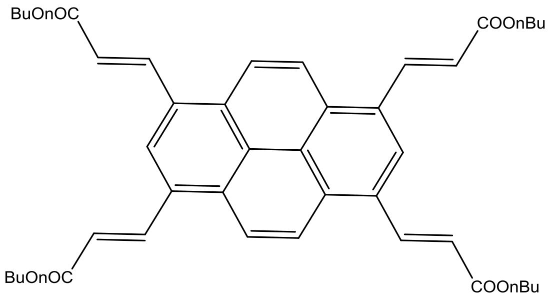


$^1$H NMR (400 MHz, CDCl3, TMS): δ 8.61 (d, J = 16. Hz, 4H), 8.34 (s, 6H), 6.68 (d, J = 16.0 Hz, 4H), 4.33 (t, J = 6.7 Hz, 8H), 1.83 – 1.76 (m, 8H), 1.58 – 1.48 (m, 8H), 1.04 (t, J = 7.4 Hz, 12H).

$^{13}$C NMR (100 MHz, CDCl3, TMS): δ 167.09, 140.73, 130.56, 130.21, 125.51, 124.49, 123.52, 122.62, 65.29, 31.29, 19.74, 14.28.

## 1,3,6,8-Tetra((E)-styryl)pyrene (4b)

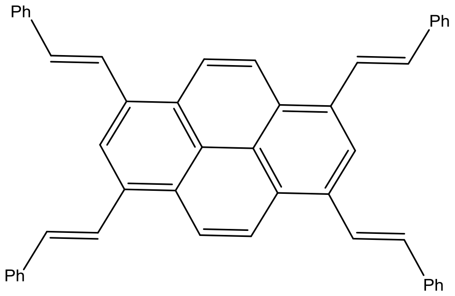


We have crystal data for this compound and it is having Solubility problem so we could not get pure NMR

## Hexabutyl 3,3',3'',3''',3'''',3'''''-(benzene-1,2,3,4,5,6-hexayl)(2E,2'E,2''E,2'''E,2''''E,2'''''E)-hexaacrylate (5a)

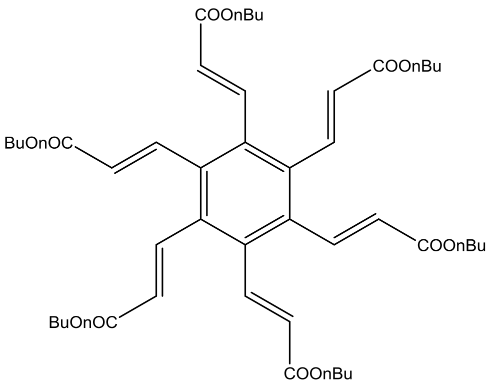

### 1,2,3,4,5,6-Hexa((E)-styryl)benzene (5b)

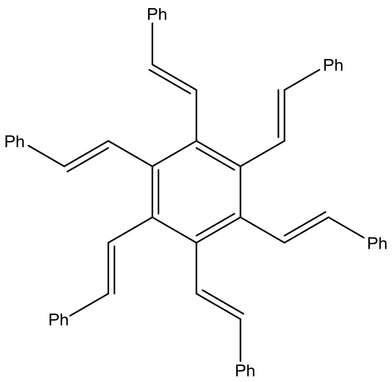


MALDI indicated the formation of the hexa substituted product in both the above cases, however, the NMR showed the presence of other isomers. This observation is similar to what has been reported in the literature for the same compound, prepared using other Pd catalysts. (S2)

(S2) Chem. Eur. J. 2005, 11, 308 – 320

### Butyl (E)-3-(4-nitrophenyl)acrylate

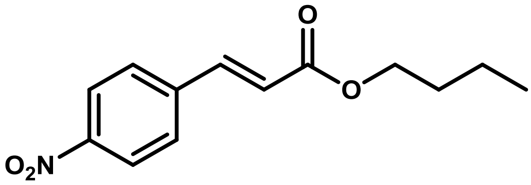


$^{1}$H NMR (400 MHz, CDCl3, TMS): δ 8.21 (d, J = 16.0 Hz, 1H), 7.66 (dd, 4H), 6.54 (d, J = 16.0 Hz, 1H), 4.20 (t, J = 7.1Hz, 2H), 1.72 – 1.62 (m, 2H), 1.47 – 1.35 (m, 2H), 0.94 (t, J = 7.4 Hz, 3H).

$^{13}$C NMR (100 MHz, CDCl3, TMS): δ 165.99, 148.31, 141.45, 128.52, 124.02, 122.46, 64.76, 30.56,

HRMS: calcd for $C_{13}H_{15}NO_4$ $[M+H]^+$ 250.1079, found 250.1076.

### Butyl (E)-3-(4-methoxyphenyl)acrylate

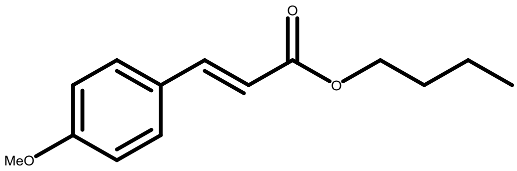


$^{1}$H NMR (400 MHz, CDCl3, TMS): δ 7.63 (d, J = 16. Hz, 1H), 7.47 (d, J = 8.8 Hz, 2H), 6.90 (d, J = 8.0 Hz, 2H), 6.31 (d, J = 16.0 Hz, 1H), 4.19 (t, J = 6.7Hz, 2H), 3.83 (s, 3H), 1.70 – 1.64 (m, 2H), 1.48-1.39 (m, 2H), 0.96 (t, J = 7.4 Hz, 3H).

$^{13}C$ NMR (100MHz, CDCl3, TMS): δ 168.18, 162.02, 144.92, 130.40, 127.92, 116.48, 115.01, 64.98, 56.08, 31.52, 19.92, 14.47.

HRMS: calcd for $C_{14}H_{18}O_3Na$ $[M +Na]^+$ 257.1153, found 257.1165.

## Butyl (E)-3-(p-tolyl)acrylate

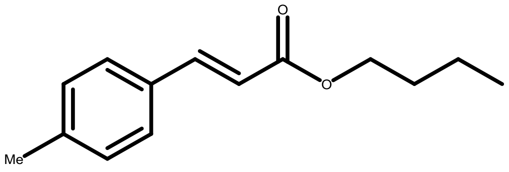


$^1H$ NMR (400 MHz, CDCl3, TMS): ) δ 7.66 (d, J = 16.0 Hz, 1H), 7.47 (d, J = 8.8 Hz 2H), 7.19 (d, J =8. Hz, 2H), 6.40 (d, J = 16.0, 1H), 4.21 (t, J = 6.7 Hz, 2H), 2.37 (s, 3H), 1.73-1.66 (m, 2H), 1.47-1.40 (m, 2H), 0.97 (t, J = 7.4Hz, 3H).

$^{13}C$ NMR (100 MHz, CDCl3, TMS): δ 167.99, 145.22, 141.28, 132.40, 130.26, 128.70, 117.85, 65.01, 31.46, 22.12, 19.87, 14.42.

HRMS: calcd for $C_{14}H_{18}O_2Na$ $[M +Na]^+$ 241.1204, found 241.1212.

## Butyl (E)-3-(4-cyanophenyl)acrylate

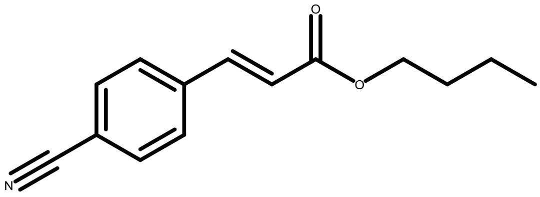


$^1H$ NMR (400 MHz, CDCl3, TMS): δ 7.48 (d, J = 16.0 Hz, 1H), 7.43 (d, 8.8 Hz, 2H ), 7.41 (d, 8.0Hz, 2H), 6.33 (d, J = 16.0 Hz, 1H), 4.03 (t, J = 6.7 Hz, 2H), 1.71 – 1.64 (m, 2H), 1.48-1.38 (m, 2H), 0.96 (t, J = 7.4 Hz, 3H).

$^{13}C$ NMR (100 MHz, CDCl3, TMS): δ 166.55, 142.43, 139.08, 132.96, 128.73, 122.21, 118.69, 113.65, 65.15, 31.02, 19.49, 14.05.

HRMS: calcd for $C_{14}H_{15}NO_2Na$ $[M +H]^+$ 230.11181, found 230.1187.

## (E)-1-Methoxy-4-styrylbenzene

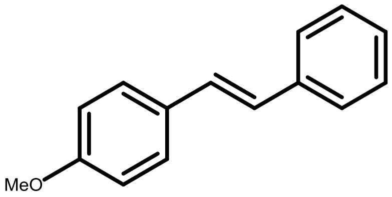

$^{1}H$ NMR (500 MHz, CDCl3, TMS): δ 7.52-7.57 (m,4H), 7.42 (d, J = 7.1 Hz, 2H), 7.32 (d, J = 7.7 Hz, 1H), 7.14 (d, J = 16.3 Hz, 1H), 7.05 (d, J = 16.3 Hz, 1H), 6.98 (d, J = 8.1 Hz, 2H), 3.90 (s, 3H).

$^{13}C$ NMR (100 MHz,CDCl3, TMS): δ = 159.3, 137.6, 130.1, 128.6, 128.2, 127.7, 127.2, 126.6, 126.2, 114.1, 55.3.

## (E)-1-Nitro-4-styrylbenzene

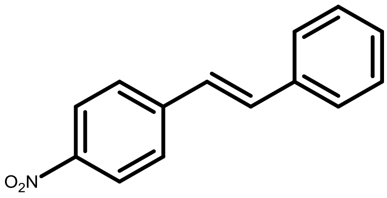


$^{1}H$ NMR (400 MHz, CDCl3, TMS): δ 8.19 (d, J = 8.9 Hz, 2H), 7.60 (d, J = 8.8 Hz, 2H), 7.52 (d, J = 7.3 Hz, 2H), 7.37 (t, J = 7.4 Hz, 2H), 7.31 (d, J = 7.2 Hz, 1H), 7.22 (d, J = 16.0Hz, 1H), 7.11 (d, J = 16.3 Hz, 1H).

$^{13}C$ NMR (100 MHz, CDCl3, TMS): δ 146.87, 143.98, 136.29, 133.43, 129.03, 127.15, 126.99, 126.40, 124.28.

HRMS: calcd for $C_{14}H_{11}NO_2Na$ $[M +H]^+$ 226.0868, found 226.0868.

## (E)-4-styrylbenzonitrile

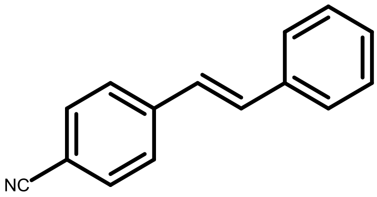


$^{1}H$ NMR (400 MHz, CDCl3, TMS): δ 7.62 (d, J = 8.4 Hz, 2H), 7.59 – 7.52 (m, 4H), 7.40 (t, J = 7.4 Hz, 2H), 7.34 (d, J = 7.2 Hz, 1H), 7.21 (d, J = 16.3 Hz, 1H), 7.08 (d, J = 16.3 Hz, 1H).

$^{13}C$ NMR (100 MHz, CDCl3, TMS): δ 142.01, 136.46, 132.67, 132.58, 129.06, 128.85, 127.06, 126.89, 119.26, 110.71.

HRMS: calcd for $C_{15}H_{11}NH$ $[M +H]^+$ 206.0969 found 206.0970.

## 1,1'-Biphenyl

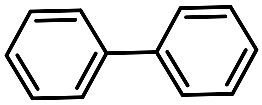

$^1$H NMR (500 MHz,CDCl3, TMS): δ 7.79 (d, J = 7.5 Hz, 4H), 7.63 (t, J = 7.4 Hz, 4H), 7.53 (d, J = 7.2 Hz, 2H).

$^{13}$C NMR (50 MHz, CDCl3, TMS): δ 141.44, 128.94, 127.39,127.37.

HRMS: calcd for $C_{12}H_{10}$ [M ]$^+$ 154.0783, found 154.0875.

## 4,4'-Dimethoxy-1,1'-biphenyl

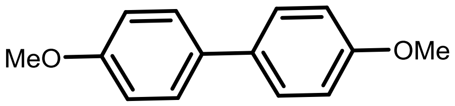


$^1$H NMR (400 MHz,CDCl3, TMS): δ 7.49 (d, *J* = 8.8 Hz, 4H), 6.97 (d, *J* = 8.8 Hz, 4H), 3.85 (s, 6H).
$^{13}$C NMR (50 MHz, CDCl3, TMS): δ 158.74, 133.52, 127.76, 114.21, 55.37

HRMS: calcd for $C_{14}H_{14}O_2NH$ [M +H]+ 215.1072, found 215.1072.

## Dimethyl [1,1'-biphenyl]-4,4'-dicarboxylate

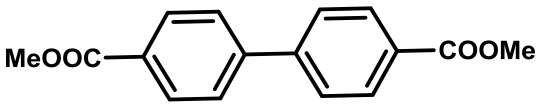


$^1$H NMR (400 MHz, CDCl3, TMS): δ 8.12 (d, J = 8.0 Hz, 4H), 7.68 (d, J = 8.0 Hz, 4H), 3.94 (s, 6H).

$^{13}$C NMR (100 MHz, CDCl3, TMS): δ 166.88, 144.41,130.28, 129.75, 127.32, 52.31.

HRMS: calcd for $C_{16}H_{14}O_4Na$ [M +Na]+ 271.097, found 271.0966.

## 1,1'-([1,1'-Biphenyl]-4,4'-diyl)bis(ethan-1-one)

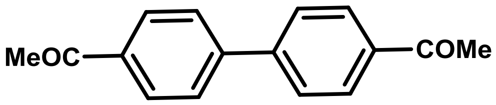


$^1$H NMR (400 MHz, CDCl3, TMS): δ 8.06 (d, J = 8.0 Hz, 4H), 7.72 (d, J = 8.0 Hz, 4H), 2.65 (s, 6H).

$^{13}$C NMR (100 MHz, CDCl3, TMS): δ 197.73, 144.42, 136.63, 129.09, 127.53, 26.80 .

HRMS: calcd for $C_{16}H_{14}O_2H$ [M +H]+ 239.1072, found 239.1071.

## Methyl [1,1'-biphenyl]-4-carboxylate

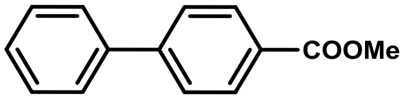


$^1$H NMR (400 MHz, CDCl3, TMS): δ 8.18 (d, J = 8.5 Hz, 2H), 7.68 (d, J = 8.4 Hz, 2H), 7.62 (d, J = 7.1 Hz, 2H), 7.48 (d, J = 7.1 Hz, 2H), 7.40 (t, J = 7.3 Hz, 1H), 3.97 (s, 1H).

$^{13}$C NMR (100 MHz, CDCl3, TMS): δ 165.89, 147.11, 140.46, 131.31, 129.63, 128.99, 128.30, 127.98, 127.85, 80.68.

## 1-([1,1'-Biphenyl]-4-yl)ethan-1-one

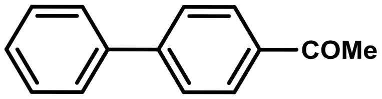


$^1$H NMR (400 MHz, CDCl3, TMS): δ 8.04 (d, J = 8.4 Hz, 2H), 7.69 (d, J = 8.5 Hz, 2H), 7.66 – 7.61 (m, 2H), 7.48 (t, J = 7.4 Hz, 2H), 7.41 (d, J = 7.3 Hz, 1H), 2.64 (s, 3H).

$^{13}$C NMR (100 MHz, CDCl3, TMS): δ 197.62, 145.59, 139.66, 135.63, 128.76, 128.73, 128.04, 127.07, 127.03, 26.48.

## 4-Nitro-1,1'-biphenyl

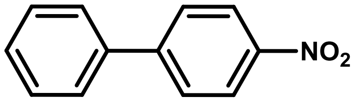


$^1$H NMR (400 MHz, CDCl3, TMS): δ 8.29 (d, J = 8.0 Hz, 2H), 7.73 (d, J = 8.0 Hz, 2H), 7.63 (d, J = 8.0 Hz, 2H), 7.55 – 7.40 (m, 3H).

$^{13}$C NMR (100 MHz, CDCl3, TMS): δ 147.69, 147.13, 138.81, 129.25, 129.02, 127.86, 127.46, 124.18.

## 4-Methoxy-1,1'-biphenyl

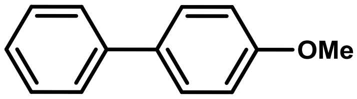


$^1$H NMR (400 MHz, CDCl3, TMS): δ 7.58 (m, 4H), 7.45 (t, J = 8.0 Hz, 2H), 7.37 (d, J = 8.0 Hz, 1H), 7.02 (d, J = 8.6 Hz, 2H), 3.87 (s, 3H).

$^{13}$C NMR (100 MHz, CDCl3, TMS): δ 159.66, 141.33, 134.27, 129.26, 128.67, 127.25, 127.19, 114.72, 55.83.

## *Single Crystal structures of some of the compounds that were isolated as products from the Heck or C-C coupling reactions carried out in this study.*

### *Single crystal structure determination:*

Single-crystal data was collected on a Bruker SMART APEX four-circle diffractometer equipped with a CMOS photon 100 detector (Bruker Systems Inc.) and with a Cu Kα radiation (1.5418 Å). The incident X-ray beam was focused and monochromated using Microfocus (IµS). Crystal of the product (e.g. compound 9 of table s4) was mounted on nylon Cryo loops with Paratone-N oil. Data was collected at 173(2) K. Data was integrated using Bruker SAINT software and was corrected for absorption using SADABS. Structure was solved by Intrinsic Phasing module of the Direct methods and refined using the SHELXTL 97 software suite. All non-hydrogen atoms were located from iterative examination of difference F-maps following which the structure was refined using least-squares method. Hydrogen atoms were placed geometrically and placed in a riding model.

**Table s4.** In some cases the products formed using Pd-trzn-COF as catalyst were isolated as pure Single Crystals and further conformed SXRD. Few representative products have been shown below

| S.No | Structure | Name | Table |
|---|---|---|---|
| 1 | 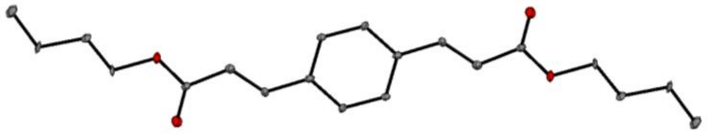 | Dibutyl 3,3'-(1,4-phenylene)(2E,2'E)-diacrylate | 1-2a |
| 2 | 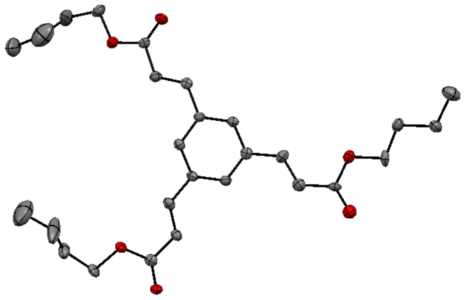 | Tributyl 3,3',3''-(benzene-1,3,5-triyl)(2E,2'E,2''E)-triacrylate | 1-3a |
| 3 | 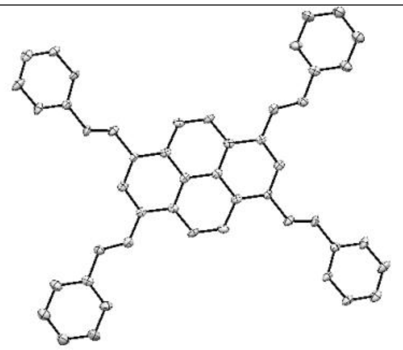 | 1,3,6,8-Tetra((E)-styryl)pyrene | 1-4a |
| 4 | 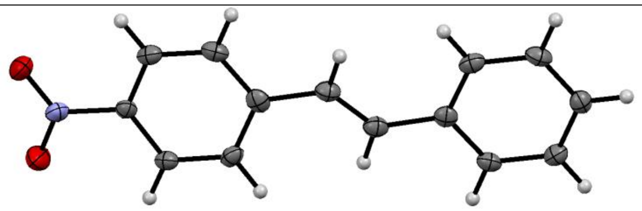 | (E)-1-Nitro-4-styrylbenzene | s1- 9 |
| 5 | 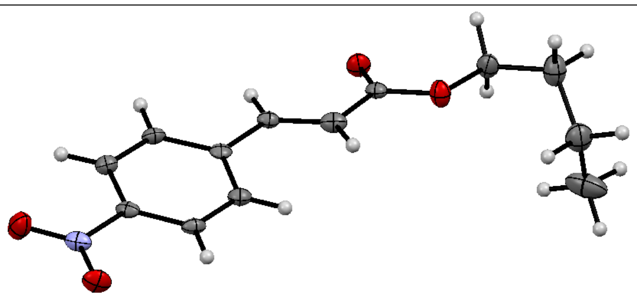 | Butyl (E)-3-(4-nitrophenyl)acrylate | s1- 5 |

| | | | |
|---|---|---|---|
| **6** | 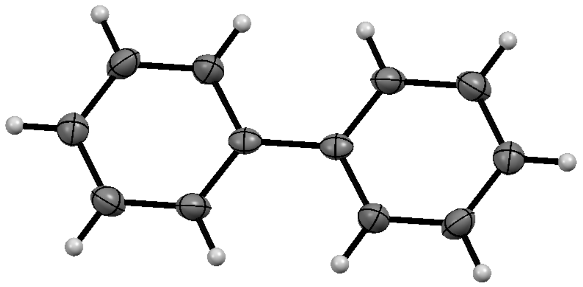 | 1,1'-Biphenyl | s2-1 |
| **7** | 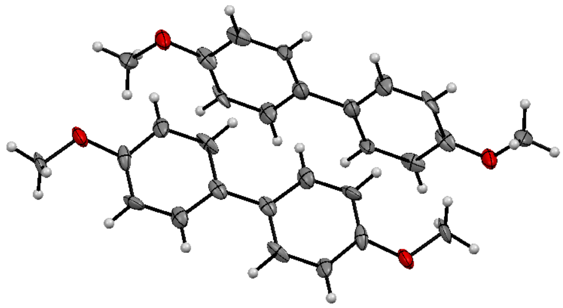 | 4,4'-Dimethoxy-1,1'-biphenyl | s2-4 |
| **8** | 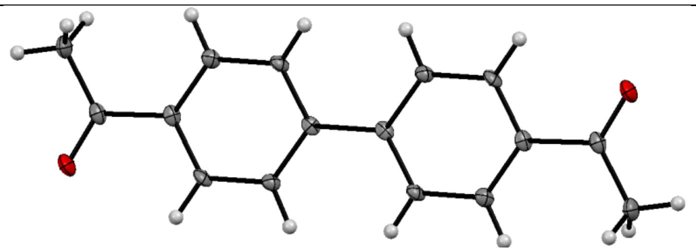 | 1,1'-([1,1'-Biphenyl]-4,4'-diyl)bis(ethan-1-one) | s2-5 |
| **9** | 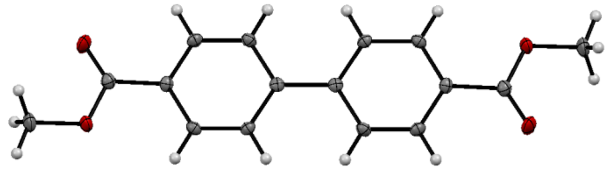 | Dimethyl [1,1'-biphenyl]-4,4'-dicarboxylate | s2-6 |

Note: The ORTEP plots for the products have been shown with a 50% probability.

# $^{1}H$ and $^{13}C$ NMR Spectra of Compound (2a)

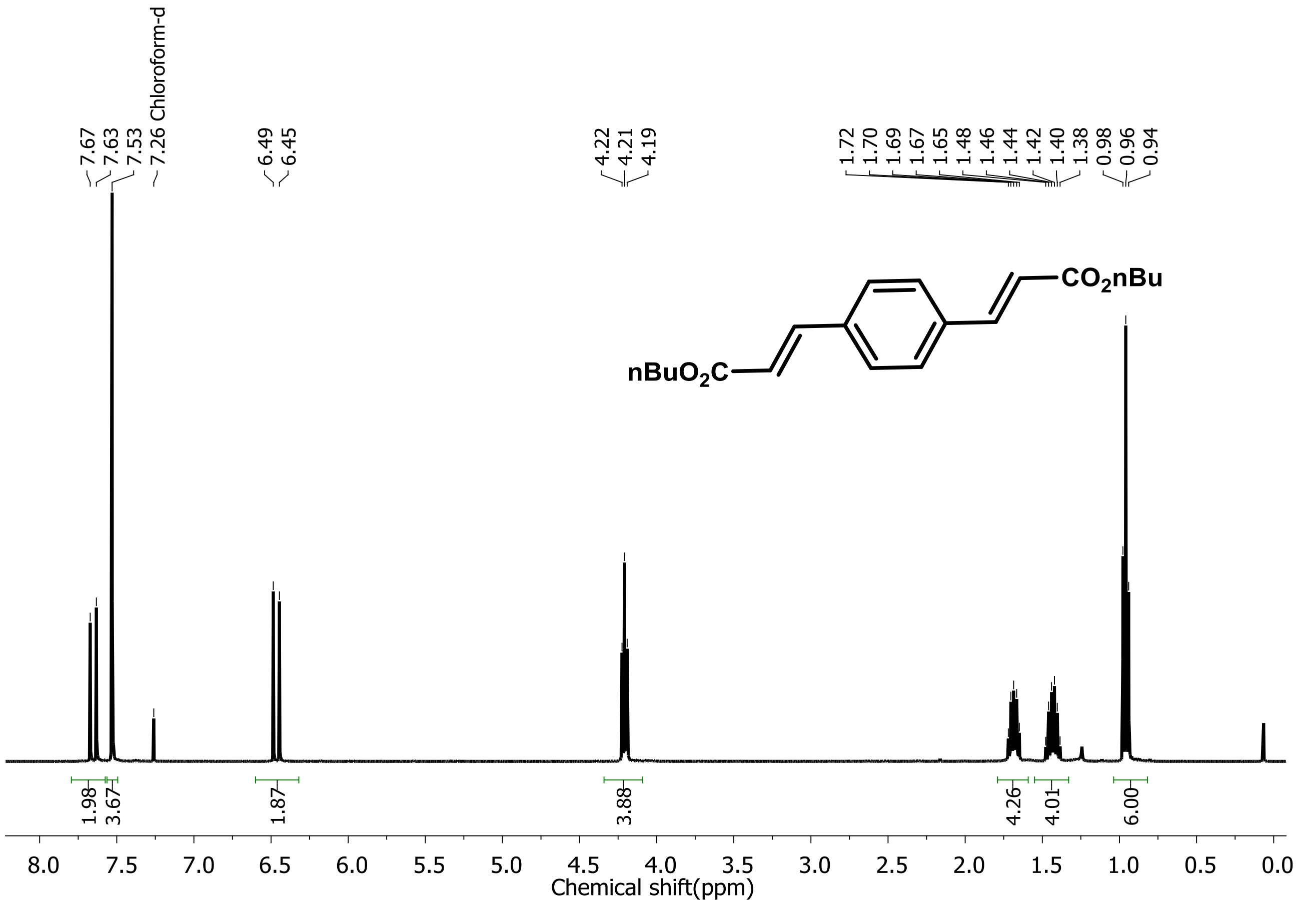


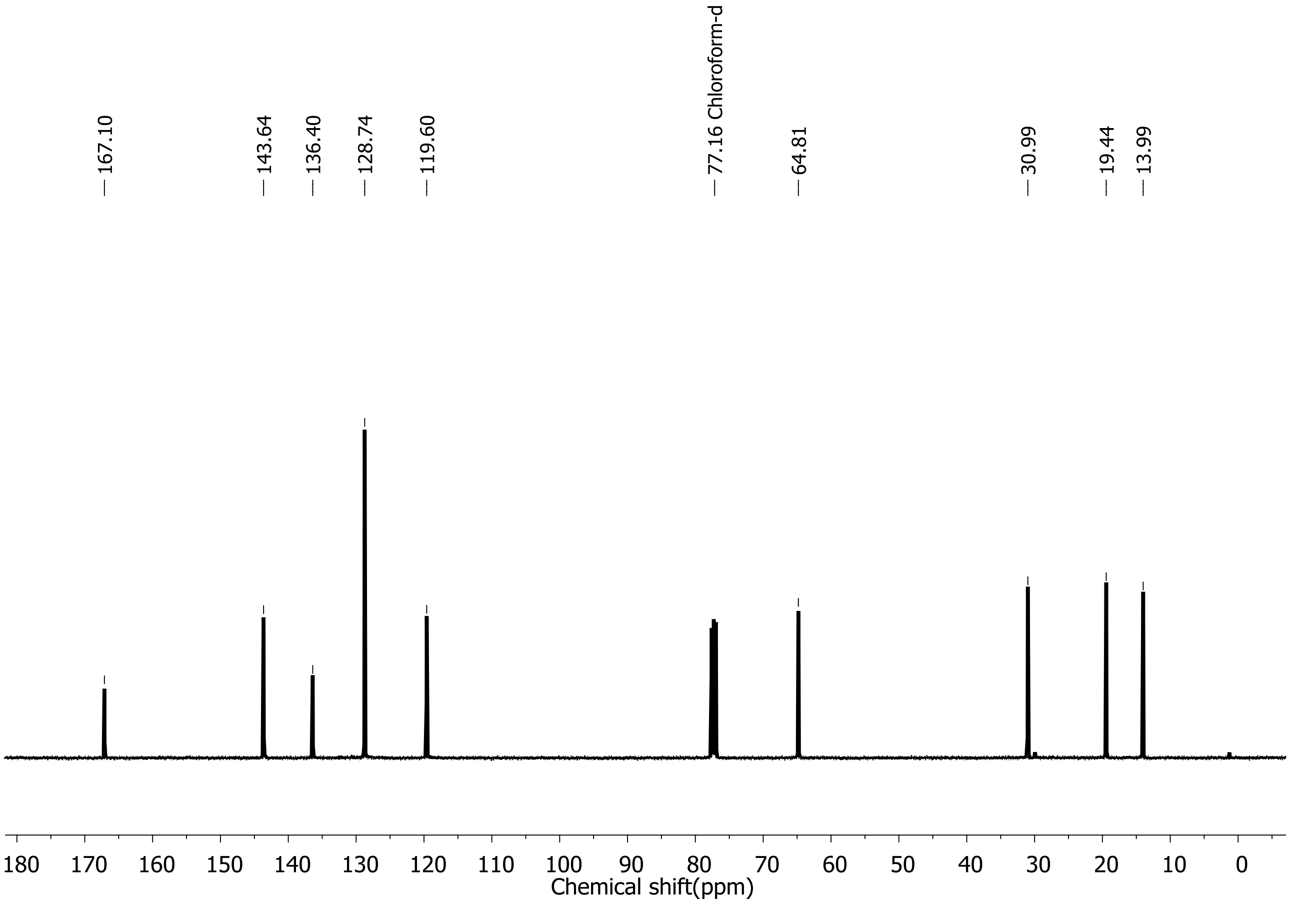

**DEPT (135) NMR Spectrum of Compound 2a**

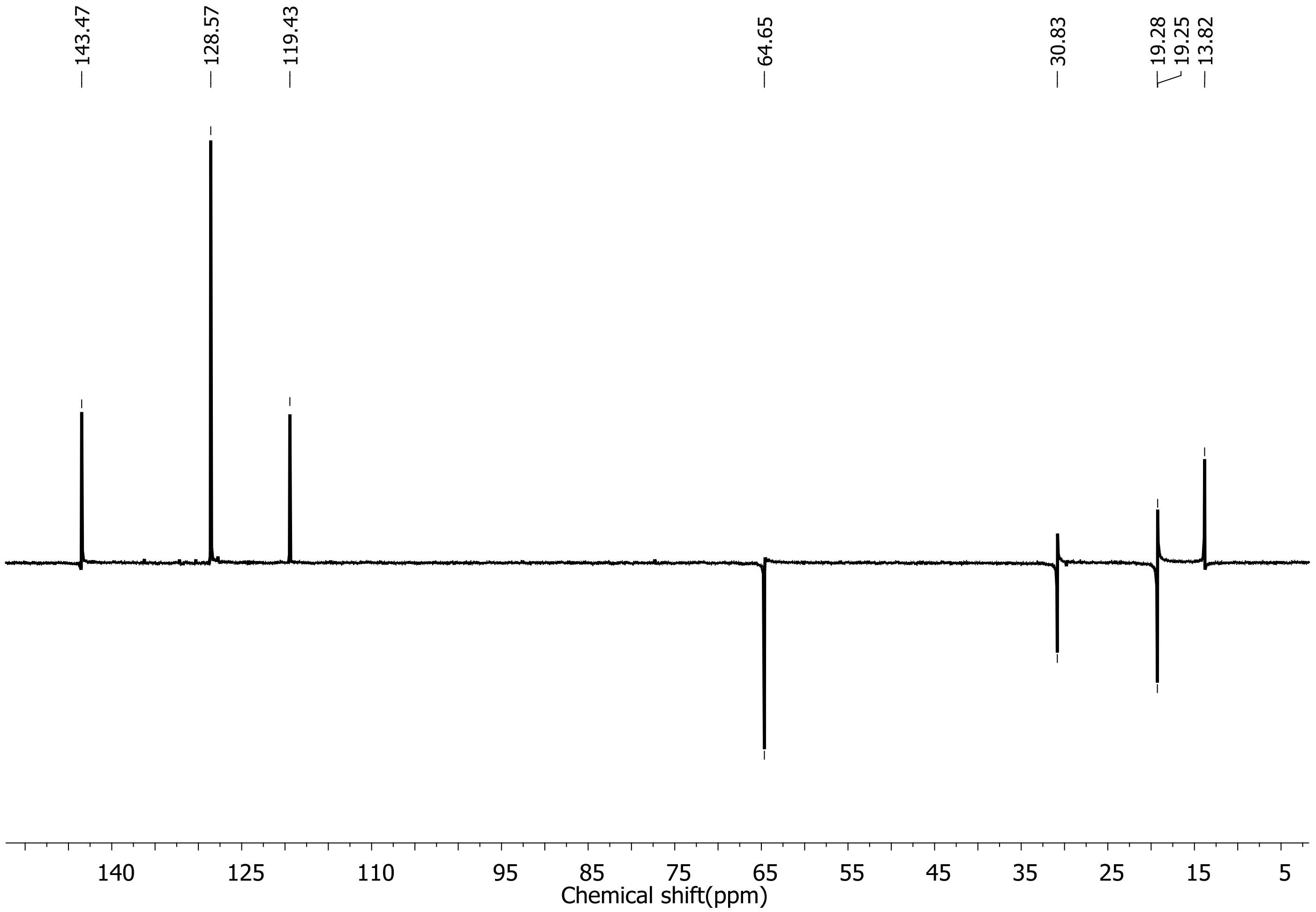

# $^{1}H$ and $^{13}C$ NMR Spectra of Compound (3a)

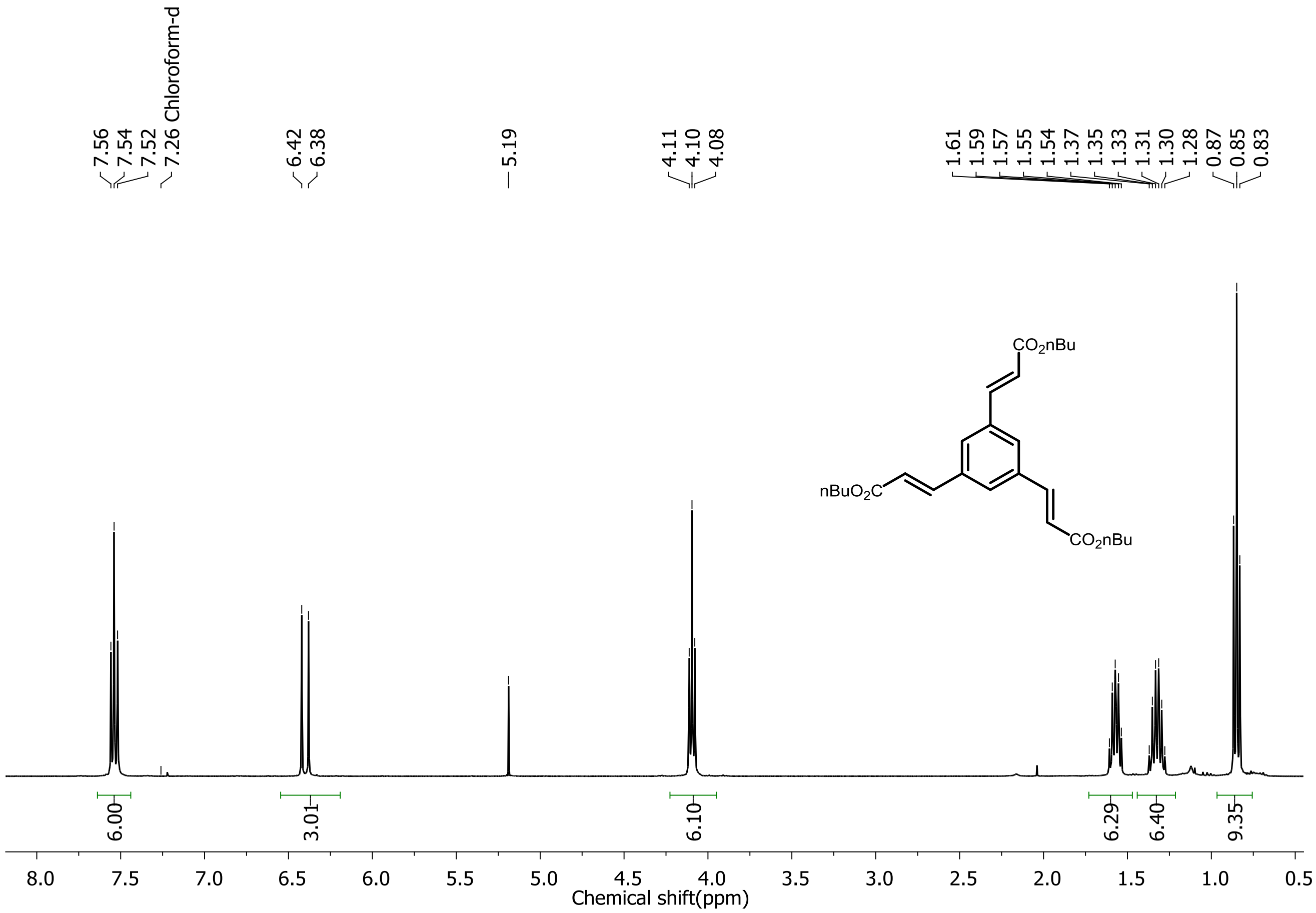


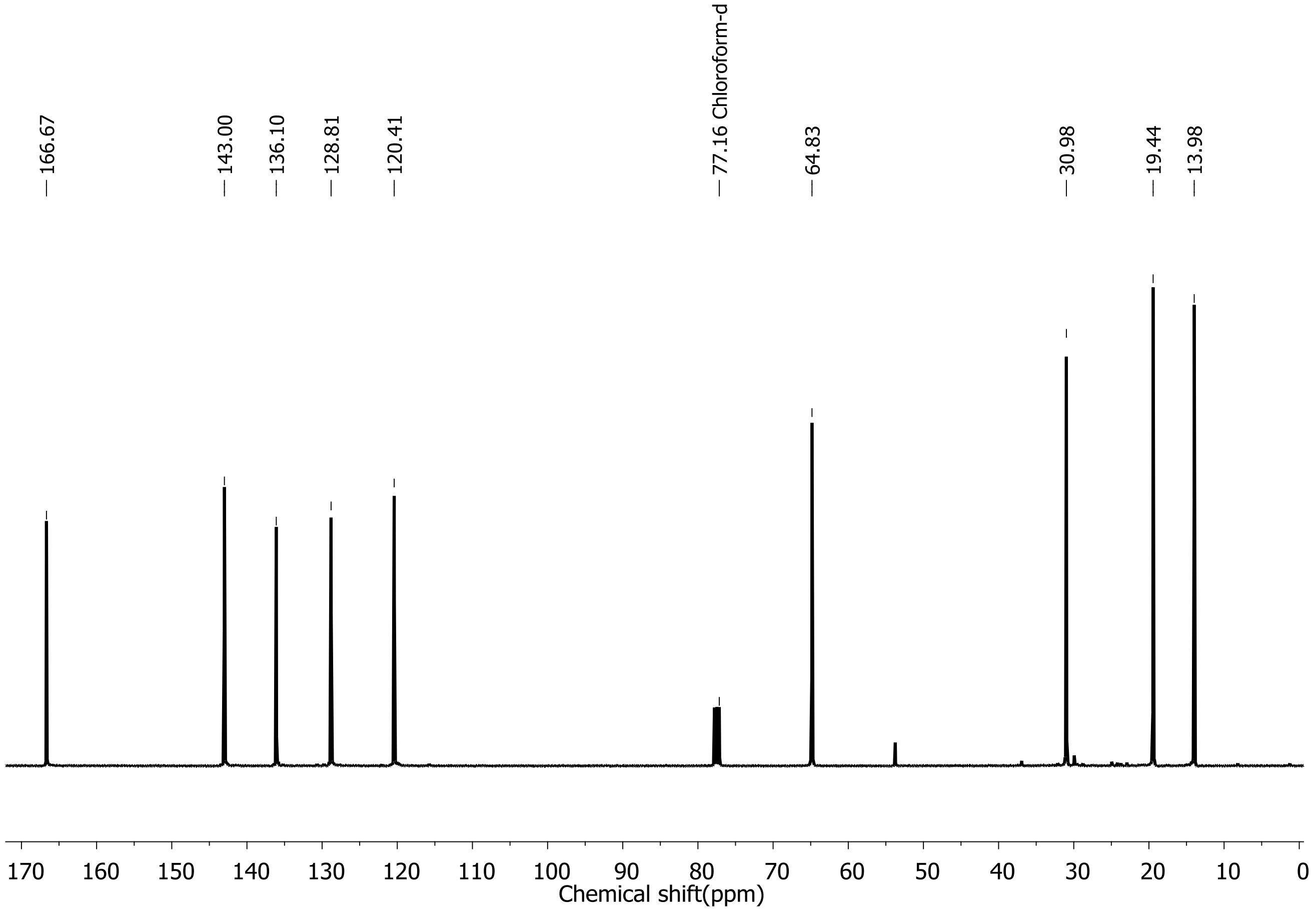

**DEPT (135) NMR Spectrum of Compound 2a**

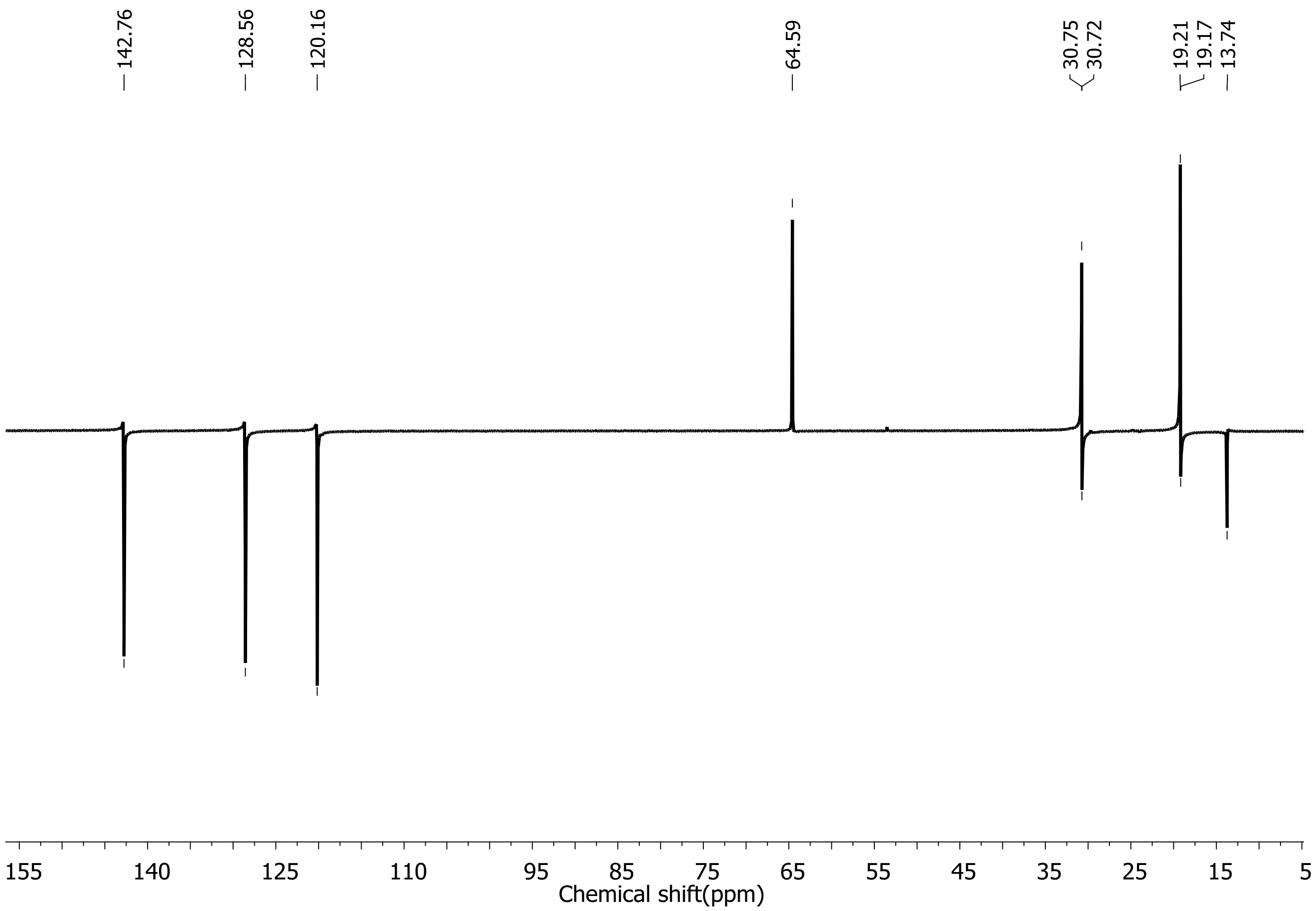

# $^{1}H$ and $^{13}C$ NMR Spectra of Compound (4a)

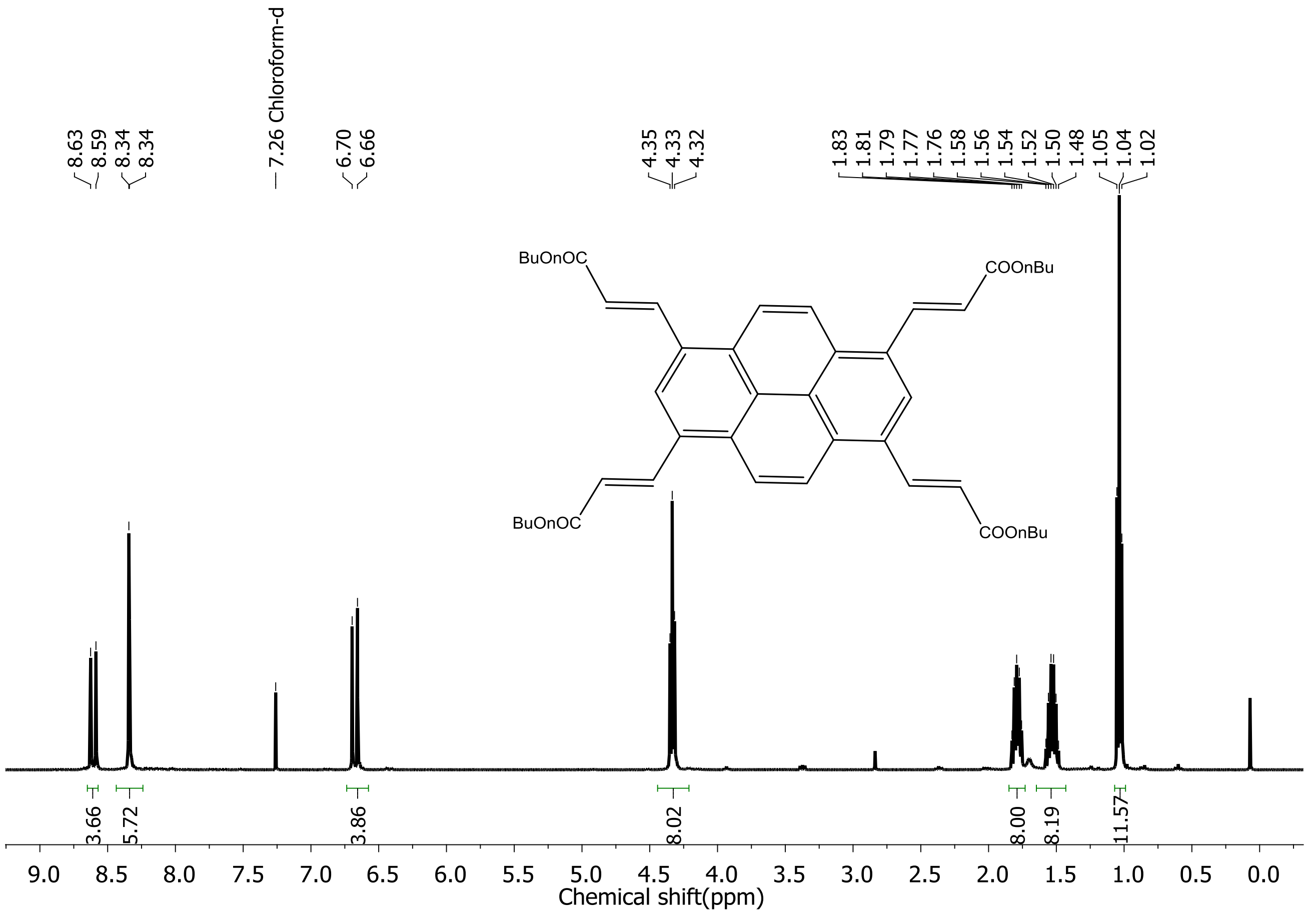


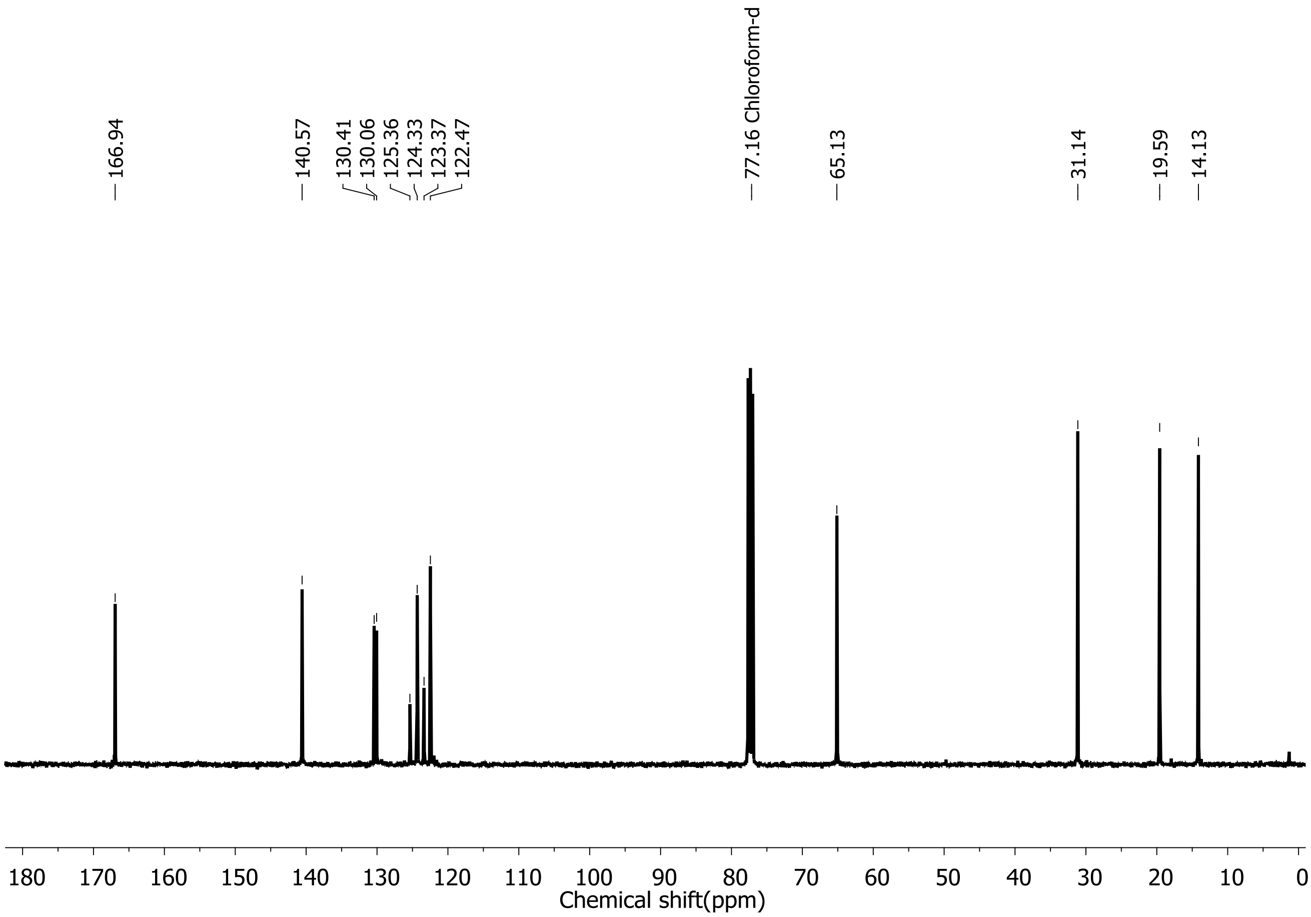

**DEPT (135) NMR Spectrum of Compound 4a**

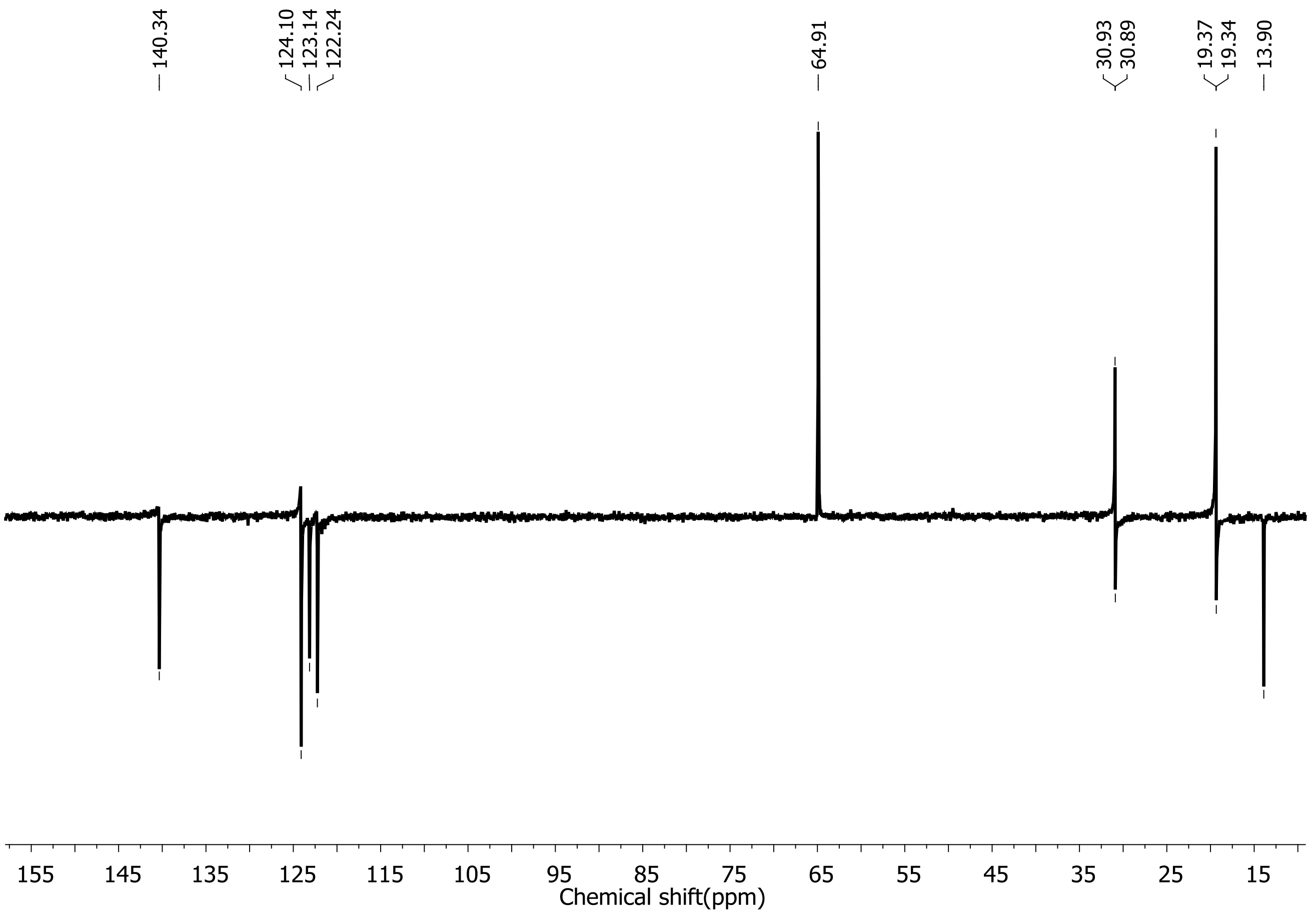

Supplement: Supplementary Information [file srep10876-s1.pdf]
